# Secreted proteins from carotid endarterectomy: an untargeted approach to disclose molecular clues of plaque progression

**DOI:** 10.1186/1479-5876-11-260

**Published:** 2013-10-16

**Authors:** Silvia Rocchiccioli, Gualtiero Pelosi, Silvia Rosini, Michele Marconi, Federica Viglione, Lorenzo Citti, Mauro Ferrari, Maria Giovanna Trivella, Antonella Cecchettini

**Affiliations:** 1 National Research Council, Institute of Clinical Physiology, Via Moruzzi, Pisa, Italy; 2 University of Pisa, Unit of Vascular Surgery, Via Paradisa, Pisa, Italy; 3 University of Pisa, Department of Clinical and Experimental Medicine, Via Volta, Pisa, Italy

**Keywords:** Atherosclerosis, Carotid plaques, Proteomics, Secretome

## Abstract

**Background:**

Atherosclerosis is the main cause of morbidity and mortality in Western countries and carotid plaque rupture is associated to acute events and responsible of 15-20% of all ischemic strokes. Several proteomics approaches have been up to now used to elucidate the molecular mechanisms involved in plaque formation as well as to identify markers of pathology severity for early diagnosis or target of therapy. The aim of this study was to characterize the plaque secretome. The advantage of this approach is that secretome mimics the *in vivo* condition and implies a reduced complexity compared to the whole tissue proteomics allowing the detection of under-represented potential biomarkers.

**Methods:**

Secretomes from carotid endarterectomy specimens of 14 patients were analyzed by a liquid chromatography approach coupled with label free mass spectrometry. Differential expression of proteins released from plaques and from their downstream distal side segments were evaluated in each specimen. Results were validated by Western blot analysis and ELISA assays. Histology and immunohistochemistry were performed to characterize plaques and to localise the molecular factors highlighted by proteomics.

**Results:**

A total of 463 proteins were identified and 31 proteins resulted differentially secreted from plaques and corresponding downstream segments. A clear-cut distinction in the distribution of cellular- and extracellular-derived proteins, evidently related to the higher cellularity of distal side segments, was observed along the longitudinal axis of carotid endarterectomy samples. The expressions of thrombospondin-1, vitamin D binding protein, and vinculin, as examples of extracellular and intracellular proteins, were immunohistologically compared between adjacent segments and validated by antibody assays. ELISA assays of plasma samples from 34 patients and 10 healthy volunteers confirmed a significantly higher concentration of thrombospondin-1 and vitamin D binding protein in atherosclerotic subjects.

**Conclusions:**

Taking advantage of the optimized workflow, a detailed protein profile related to carotid plaque secretome has been produced which may assist and improve biomarker discovery of molecular factors in blood. Distinctive signatures of proteins secreted by adjacent segments of carotid plaques were evidenced and they may help discriminating markers of plaque complication from those of plaque growth.

## Background

Atherosclerosis is a chronic immune-inflammatory disease of the wall of medium- and large-sized arteries and it is the main cause of morbidity and mortality in Western countries due to acute cardiovascular events secondary to partial or total thrombotic obstruction of vessel lumen [1,2]. Rupture of carotid atherosclerotic plaques leads to atherothrombosis that accounts for approximately 20% of all strokes, a leading cause of disability, and the third most common cause of death [3].

Recently, a great interest has been focused on the identification of tissue markers of atherosclerosis, at genomics, transcriptomics [4] or proteomics levels. The majority of proteomics studies were performed on plaque extracts analyzed by two-dimensional electrophoresis followed by MALDI TOF mass spectrometry [5,6], but, with this approach, mainly constitutive proteins are identified and potential, usually low-expressed biomarkers may not be detected. To overcome these limitations, Olson et al. [7] applied two-dimensional differential gel electrophoresis (2D-DIGE) in combination with tandem mass spectrometry (MS/MS) to compare protein distribution in an intra-individual study of severely atherosclerotic segments of internal carotid artery compared to partially preserved segments. In this way, they identified 19 proteins with a differential distribution along the artery. Another interesting approach was exploited by Martinet et al. [8] who screened human carotid endarterectomy (CEA) specimens using 823 monoclonal antibodies with Western array technology and were able to identify 7 differentially expressed proteins. The potential use of tissue proteomics as a tool for clinically useful biomarker discovery has been recently prospected by de Kleijn et al. [9] who found that osteopontin expression in carotid plaques and in blood is predictive for atherothrombotic events but it does not correlate with vulnerability features.

An alternative proteomics approach consists of the analysis of proteins secreted by explanted arteries and was first suggested by Duran et al. [10]. The great advantage of tissue secretome studies is that secretome mimics the *in vivo* condition and implies a reduced complexity compared to serum/plasma or entire tissue proteomics as well as a much narrower protein dynamic range, thus allowing the detection of under-represented potential biomarkers.

In fact, in biomarker discovery, plasma represents the sample of choice since it shows traces of all biological events and, moreover, it can be easily and non-invasively collected. On the contrary, in proteomics studies, plasma proteome is hampered by major limits such as the high dynamic range of plasma proteins and a great biological variability. For all these reasons, the analysis of proteins that are secreted by tissues into circulation has attained interest for discovery of novel biomarkers and it represents a way to gain knowledge of biological mechanisms [10,11].

An optimal culture set-up of arterial secretome in order to reduce plasma contamination and detect low abundance proteins is a recent achievement [12]. Also recently, secretomes from thromboendartectomy specimens were exploited to select nine secretome-specific antibodies that allowed the immuno-purification and successive identification of 22 proteins. Among them, junction plakoglobin has been suggested as a potential biomarker of atherosclerosis [13].

A main issue in molecular studies of vascular pathology is the cellular and extracellular heterogeneity of the plaque and of the adjacent wall where multiple components (calcium, lipids, collagens and others) and cell types (vascular smooth muscle cells (VSMCs), endothelial cells (EC), macrophages and other inflammatory cells) are present, all contributing to plaque progression and/or complication.

Several previous and recent papers have highlighted a close link between the longitudinal distribution of mechanical forces (flow shear stress and plaque wall stress) and corresponding morphological features (cell distribution and type) along plaque and its distal side [14-18]. In particular, the low flow shear stress in downstream side is associated to atherosclerosis progression with increased VSMCs and macrophages, whereas the high plaque wall stress in the upstream area is associated to cap rupture of vulnerable lesions and increased expression of proteolysis and apoptosis markers [14]. These reports support the opinion that carotid plaque and its corresponding adjacent distal side may retain distinctive protein signatures: therefore, differential expression of proteins released by plaque-containing upstream segment (P) and by its downstream distal side (DS) segment has been evaluated in each CEA specimen.

Aims of the study were: (a) to characterize the overall atherosclerotic carotid secretion with an untargeted approach able to reconstruct a complete protein map; (b) to evaluate the differences in secretomes from central plaque and corresponding distal side segments, as putative areas of plaque complication and stable growth respectively; (c) to conduct a secretome-assisted plasma analysis of some differentially expressed proteins to be exploited as markers of disease for non-invasive risk assessment.

Secretomes were analyzed by a liquid chromatography approach coupled with mass spectrometry (LC-MS/MS). Label-free MS/MS based quantification was performed and validated by Western blot analysis and ELISA assay. Histology and immunohistochemistry were performed to characterise CEA specimens and to localize the molecular factors highlighted by proteomics. ELISA assays of thrombospondin-1 and vitamin D binding protein were performed in plasma samples of 34 CEA candidates and 10 healthy controls.

## Methods

### Ethics statement

Clinical data were derived from medical records, following data security guidelines and declaration of Helsinki. All subjects gave written informed consent to participate to the study in accordance with the Ethics Committee requirements. The ethical approval for this study was granted by Ethics Commission (Clinical Trials Ethics Commission) of Pisa University Hospital.

### Patient clinical characterization

Human internal carotid artery (ICA) specimens and pre surgery plasma samples from 10 males and 4 females undergoing CEA for symptomatic or asymptomatic stenosis ≥70% were obtained from the Vascular Surgery Unit of Pisa University Hospital and immediately processed at the Institute of Clinical Physiology. Additional 20 plasma samples of CEA candidates and 10 plasma samples of healthy volunteers were collected at the Vascular Surgery Unit of Pisa University Hospital and at the Institute of Clinical Physiology respectively.

Patient characteristics and database information on smoking habit, hypercholesterolemia and diabetes are summarized in Table 1.

**Table 1:** Clinical characteristics of population

**Feature**		**Value (±SD)**	**Value (±SD)**	**Value (±SD)**
		**CEA patients for secretome (N=14)**	**CEA patients for plasma validation (N=34)**	**Healthy volunteers (N=10)**
Gender	Females (n)	4	10	4
	Males (n)	10	24	6
Age (years±SD)		72±9	74±7	70±5
Symptomatic (n)		6	11	-
Type of event	Stroke (n)	2	3	-
	TIA (n)	4	8	-
Asymptomatic (n)		8	23	-
Stenosis of ICA (%±SD)		80±9^*^	79±8^*^	-
Diabetes (n)		6	9	-
Hypertension (n)		10	27	1
Smoking (n)		4	13	1
Statin treatment (n)		10	21	-
AntiaggregantTherapy (n)		14	32	-
Hypercholesterolemia (n)		4	18	2

Categorical values are given as number of patients (n).

Continuous variables are given as mean±SD.

TIA: Transient Ischemic Attack. ICA: Internal Carotid Artery.

*Stenosis was measured by carotid EchoColor Doppler.

Symptomatic patients were classified as CEA candidates who presented with TIA or stroke within 6 months before surgery.

No differences between symptomatic and asymptomatic patients were found for any of the reported parameters. Smokers were classified as individuals who smoked at least three cigarettes per day at the time of analysis, past smokers had quit smoking for at least 6 months, and no-smokers were individuals who had never smoked. Smoker patients were the combined group of the past- and the current-smokers.

Stenosis on ICA was ≥ 70% at carotid Echocolor Doppler in all patients, whose CEA specimen secretomes were analysed (mean 80±9%), without significant differences between asymptomatic and symptomatic (79±8 vs 82±10 %).

### Tissue processing and secretome preparation

The arterial tissue was cultured according to de la Cuesta et al. [12] with modifications. CEA specimens (n=14) from surgery were transported in PBS on ice to the laboratory. Each specimen was crosscut under macroscopic examination to separate P from DS segment. After repeated washing in PBS to remove blood traces, samples were incubated in 6-well plates in 2 ml of Eagle's Minimal Essential Medium (Sigma-Aldrich) supplemented with penicillin and streptomycin, without FBS and phenol red at 37°C in a humidified atmosphere of 5% CO_2_. After 24 h the culture medium was harvested, centrifuged at 300× g for 10 min and stored at −80°C until analysis. Samples were concentrated by centrifugal devices Amicon Ultra-3 (Millipore) following the manufacturer's recommendations. Protein concentration was determined by bicinchoninic acid (Pierce).

### Histology and immunohistochemical characterization

After 5 to 7 day fixation in 5% buffered formalin, P and DS samples were incubated in decalcifying solution (Bio-optica 05-M0300 ), washed and dehydrated in ascending alcohols series and embedded in paraffin: transverse 5 μm thick consecutive sections were cut from each paraffin block by a rotary microtome (Microm HM 300, Bio-optica). Haematoxylin and Eosin (H&E) and Masson’s trichrome stains were used for histologic analysis. For immunohistochemistry, sections were placed on positively charged slides, de-paraffinized, rehydrated and washed in distilled water; after incubation in H_2_O_2_ at room temperature, antigen retrieval was accomplished (citrate buffer pH6 in microwave for 10 min at 500W) and then sections incubated with diluted normal blocking serum. Primary antibodies anti-αSMA (alpha smooth muscle actin, clone 1A4 ADB, Serotec) as a SMC phenotype marker, anti-CD68 (mouse, clone PG-M1, Thermo Scientific, diluted 1:150) as a macrophage phenotype marker, anti-vinculin (goat polyclonal antibody diluted 1:100, Santa Cruz Biotechnology, Inc.) and anti-thrombospondin-1 (mouse polyclonal antibody diluted 1:100, Santa Cruz Biotechnology, Inc.) were applied overnight to the slides in a 4°C humid chamber.

Following 30 min biotinylated secondary antibody and 30 min Vectastain Elite ABC reagent incubation in peroxidase substrate solution (DAB), slides were counterstained with Mayer’s haematoxylin for 1 min. and mounted (Neo-Entellan Merk). Antibody binding to cells and/or to extracellular components is visible as brown or dark brown stain (DAB), negative cells are stained blue (haematoxylin counterstain).

The same steps had been previously applied to a pool of CEA specimens (from symptomatic and asymptomatic patients) which were processed en bloc and longitudinally, instead of transversely, sectioned by microtome: microscopic examination of the obtained serial sections helped to guide and standardise the splitting of fresh CEA samples, subsequently collected for secretome, into P and DS segments.

Thrombospondin-1 and vinculin double immunostaining was performed using Vectastain Elite ABC reagent incubation in phosphatase (AP) substrate solution and Permared-AP stain for thrombospondin-1 and DAKO LSAB system HRP and subsequent peroxidase substrate solution (DAB) incubation for vinculin.

All sections from each carotid segment were examined under a light microscope (Olympus BX43) at 4× to 40× original magnification and digitized by a video system (Olympus D70 camera) interfaced to Olympus Cell Sens Dimension software for image acquisition and morphometric analysis. Quantitative analysis of antibody staining within the lesional area of each sample was carried out on several microscopic fields of consecutive sections, digitized at the same light source settings, by double observer colour thresholding: reproducibility and statistical significance of results (expressed as% positive, dark brown pixels of the entire plaque area) as well as localization of different antibodies on the same region of adjacent sections was thus accomplished.

Carotid plaques were classified according to Stary’s stages for atherosclerosis, American Heart Association Committee on Vascular Lesions [19,20].

### Reduction, alkylation and digestion

100 μL of 40 mM ammonium hydrogen carbonate (pH=8) were added to 100 μL of secretome (1 μg/μL concentration). Reduction was obtained by adding 1 μL of 1 M dithiothreitol to each sample, with an incubation of 20 min at 80°C. For alkylation, 20 μL of 100 mM iodoacetamide were added to the samples and incubated for 30 min at 37°C. Digestion was performed incubating overnight with 8 μL of trypsin solution (0.25 mg/mL) at 37°C.

### LC-MS/MS analysis

Chromatographic separation of peptides was performed using an Ultimate 3000 nano-HPLC system (LC Packings, DIONEX, USA). 100 μL of filtrate were added to a solution composed by 2% ACN and 0.1% formic acid up to 200 μL of final volume. The loading pump pre-concentrated the sample in a pre-column cartridge (PepMap-100 C18 5 mm 100 A, 30 mm id × 5 mm). Chromatographic separation of peptides was performed using a C18 PepMap-100 column (15 cm × 75 mm id, LC Packings DIONEX) equilibrated at 45°C with a solvent A (water/acetonitrile 98/2 vol/vol, 0.1% formic acid) at a flow rate of 300 nL min^-1^. Runs were performed under 60 min linear gradient from 10 to 45% of solvent B (water/acetonitrile 2/98 vol/vol, 0.1% formic acid) followed by 10 min of a purge step at 95% of B before a 20 min re-equilibration step to the starting conditions. The column was directly coupled to TripleTOF™ 5600 System (AB SCIEX, Toronto, Canada), equipped with a DuoSpray™ ion source (AB SCIEX, Toronto, Canada).

Peptides eluted from chromatography were directly processed using TripleTOF™ 5600 mass spectrometer (AB SCIEX, Toronto, Canada). The mass spectrometer was controlled by Analyst® 1.6.1 software (AB SCIEX, Toronto, Canada). For positive ionization, ion source parameters were the following: the spray voltage was 3 kV, source temperature 150°C with curtain gas set at 25, GS1 10 and GS2 0 psi nitrogen flow. For information dependent acquisition (IDA) analysis, survey scans were acquired in 250 ms and 25 product ion scans were collected if exceeding a threshold of 125 counts per second (counts/s). Total cycle time was fixed to either 1.25 s. Four time bins were summed for each scan at a pulser frequency value of 11 kHz through monitoring of the 40 GHz multichannel TDC detector with four-anode/channel detection. Dynamic exclusion was set for 1/2 of peak width (∼8 s), and then the precursor was refreshed off the exclusion list.

### Protein identification and label-free comparative analysis

MS/MS data were processed with ProteinPilot™ Software (AB SCIEX, Toronto, Canada), using the Paragon™ and Pro Group™ Algorithms and SwissProt 2011 as protein database for *Homo Sapiens*. The false discovery rate (FDR) analysis was done using the integrated tools in ProteinPilot software and a confidence level of 95% was set.

The statistical comparative analysis was performed using MarkerView™ Software 1.2.1 (AB SCIEX). The ion chromatograms of high confidence peptides identified by ProteinPilot were extracted using PeakView™ Software and then MS peak areas and identifications were imported into MarkerView™ Software. Normalization of the total plaque size was obtained using a global normalization of profiles (total protein content) using Marker View 1.2 software.

Principal Component Analysis (PCA) was performed in order to evidence groupings among the data set. All profile areas were normalized. The two groups (P and DS) were compared with *t*-test using a threshold of 95% (P value=0.05) and fold change > 2.

### Western blot analysis

Secretomes were run on a 10% SDS-PAGE, separated proteins transferred onto a nitrocellulose membrane (Amersham) using a wet transfer system (Biorad). Membranes were blocked with 3% BSA in TBST for 1 h at room temperature. Primary and secondary antibodies were diluted in 3% BSA in TBST. All primary antibodies were incubated overnight at 4°C. HRP-conjugated secondary antibodies were incubated for 1 h at room temperature. The following antibodies were used: anti-vinculin goat polyclonal antibody (1:500) (catalog number sc-7648, Santa Cruz Biotechnology, Inc), anti-thrombospondin-1 mouse monoclonal antibody (1:200) (catalog number sc-73158, Santa Cruz Biotechnology, Inc), and anti-α tubulin mouse polyclonal antibody (1:5000) (catalog number T 6074, Sigma). Chemi-luminescence was detected with ECL™ detection kit (Amersham Biosciences, Uppsala, Sweden).

Densitometric quantification of photographic films was performed using Quantity One 1-D Analysis Software (Bio-Rad). Photographic films were scanned and the pixel intensities of bands was measured subtracting the pixel intensity of the background and all the signals were recorded as Optical Density (O.D.). All the O.D. were reported in graphs and the comparative analyses of the different levels of expression of vinculin and thrombospondin-1 between P and DS samples was done integrating the resulting area under curve by using Origin 7.0 (Originlab).

### ELISA assays

Dosage by double-antibody sandwich enzyme-linked immunosorbent assay (ELISA) was performed on vitamin D binding protein and thrombospondin-1.

ELISA kits were used and reagents were prepared following the manufacturer's manual. Briefly, for vitamin D binding protein (Uscn Life Science Inc, Wuhan, China) the calibration curve ranged between 10 and 0.156 ng/mL (considering a dilution for plasma samples of 200000-fold) and the calibrator diluent was used as the zero standard. All standards and samples were assayed in duplicates.

For thrombospondin-1 assay (Bio Medical Assay, Beijing, China) the calibration curve ranged between 20 and 0.312 ng/μL. Plasma samples were diluted 100 fold.

The ELISA assay for Vitamin D binding protein (Quantikine® ELISA Human Immunoassay- USA & Canada R&D Systems, Inc.) was performed also on secretome samples without dilution. The calibration curve ranged between 1000 ng/ml and 15.5 ng/ml. The absorbance was read at 450 nm with a Fluorstar (Omega) microplate reader (Molecular Devices, Sunnyvale, CA).

### Statistical analysis

Principal component analysis (PCA) was conducted on mass spectrometric data of samples using Marker View 1.2 software.

Student’s *t* Test was used as statistical parameter between the means of continuous variables to determine significant differences between categories of mass spectrometric data. P value < 0.05 were considered significant to validate differences between categories and fold change > 2.

Statistical analyses of other data were conducted using Origin 7.0 software. Data are expressed as the mean ± SD. Differences between the means of the 2 continuous variables were evaluated by the Student’s *t* Test and results accepted when *t* Test>95% and P value<0.05. Paired *t* test was used for quantitative immunohistochemistry and ELISA assay.

### Ingenuity pathway analysis (IPA)

IPA (http://www.ingenuity.com/products/pathways_analysis.html) was performed on a restricted and selected number of identified proteins (n=56) from the secretomes of CEA specimens. The subset of chosen proteins was constituted of 34 extracellular matrix proteins, 9 plasma membrane proteins and 13 intracellular proteins. This subset of proteins was related to five function/disease pathways: migration of cells (n=39), proliferation of smooth muscle cells (n=9), vascular disease (n=11), atherosclerotic lesion (n=8) and angiogenesis (n=22). Colored points were used to evidence differentially released proteins between P and DS.

## Results

### Morphological characterization

**Figure 1:**
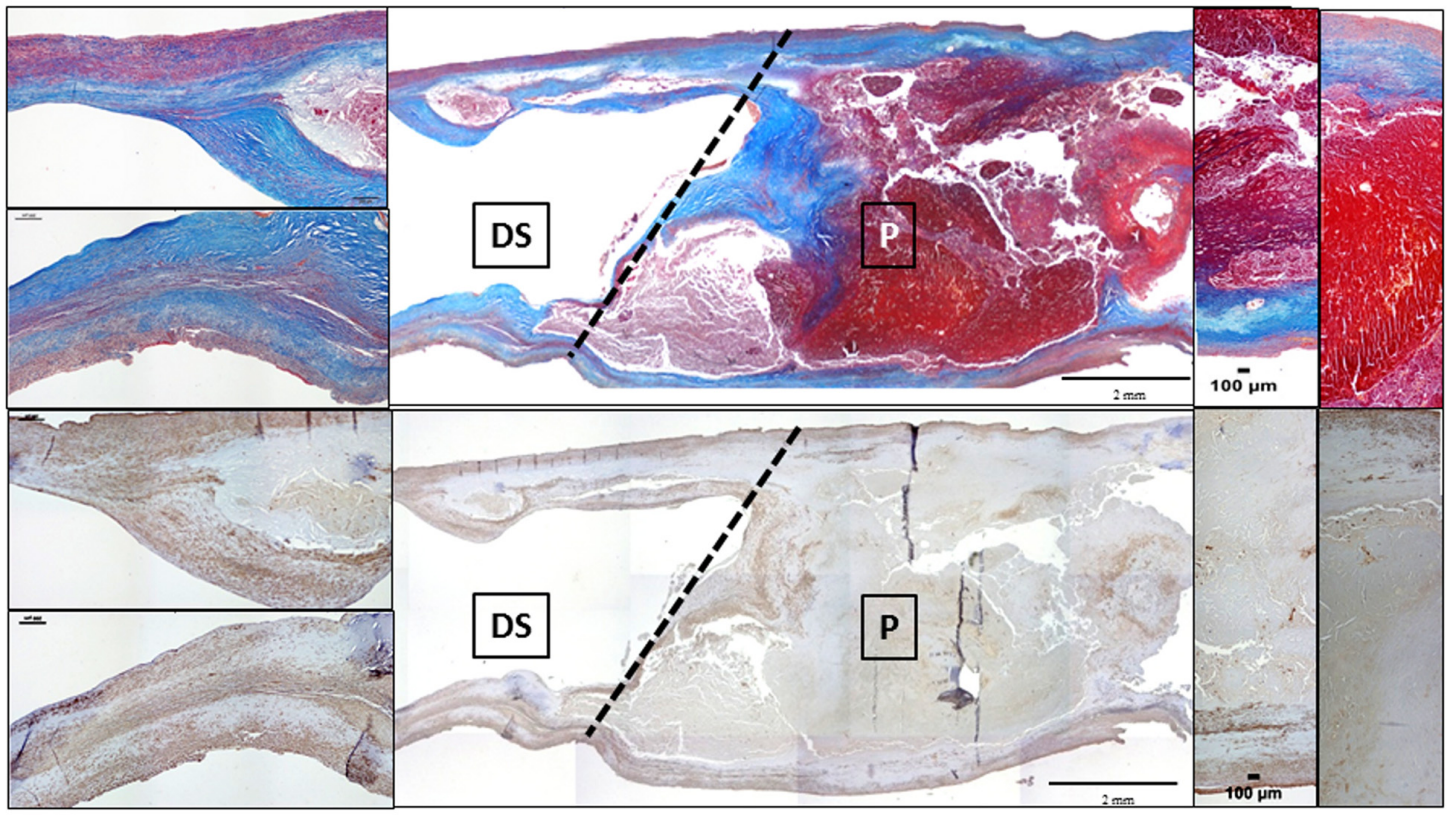
**Longitudinal section of an undivided CEA specimen at artery center line level showing the cutting line for P and DS segments separation.** Prevalent complicated plaque features of P (lipid-necrotic core, calcium deposits, fibrosis and haemorrage) and milder changes of its downstream side DS (prevalent VSMCs and collagen component, small lipid and calcium deposits) are evidenced by Masson’s trichrome and α-SMA immunostaining (from top to bottom). Original magnification 2×, insets 10×.

Microscopic examination of longitudinal sections of undivided CEA specimens evidences a plaque core containing area (P) comprised of typical components (lipid-rich necrotic core, fibrous cap, cholesterol clefts, large calcium deposits, intraplaque haemorrage/ fresh thrombi) and the distal side segment (DS) with fibromuscular tissue, small extracellular lipid and calcium deposits. Fresh CEA specimens for secretome were subsequently divided to separate central plaque P from its distal side DS, as depicted in Figure 1.

Sections from all CEA specimens, each cut in two (P and DS) were examined and classified for the presence of histologic hallmarks of atherosclerosis, including lipid-rich necrotic core, cholesterol clefts, fibrosis, calcium, thin fibrous caps, macrophage infiltrate, neovessels, intraplaque haemorrhage/thrombi. The DS segments, although free of grossly evident changes, showed small lesions at histologic examination, with atherosclerotic features similar to those classified as Stary’s types III and IV. Proximal P segments, on the other hand, showed grossly evident fibrolipidic and/or fibrocalcific, mostly complicated plaques classified as types V and VI (Table 2) [19,20].

**Table 2:** Histologic characterization of P and DS samples

	**P (% specimens)**	**DS (% specimens)**
TFCA(1)	33	0
Ca(2)	65	10
NC-Chol(3)	75	20
He(4)	35	0

(1) TFCA= thin fibrous cap, (2) Ca = calcification, (3) NC-Chol=necrotic core cholesterol clefts.

(4) He = intraplaque hemorrhage/thrombus.

**Figure 2:**
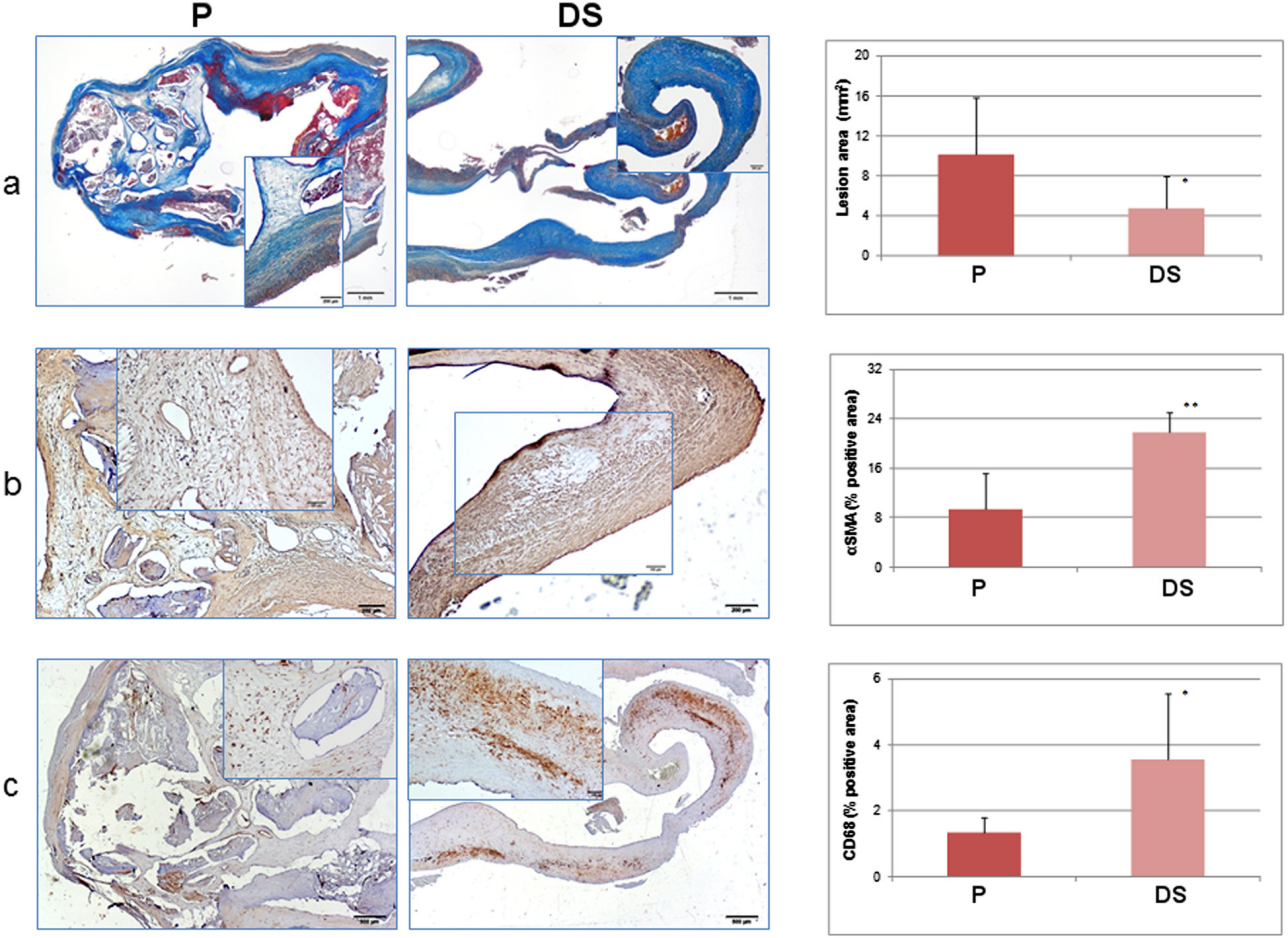
**Left: P and DS sections stained with (a) Masson’s trichrome stain, (b) α-SMA and (c) CD68 immunostain (original magnification 2×, insets 10×).** P is a type VI plaque with a necrotic core, calcifications, intraplaque thrombus and a thin fibrous cap composed of collagen and αSM-actin positive cells; minor intraplaque CD68 positive staining is present and α-SMA -actin positive contractile VSMCs are also visible on the outer border of the specimen (opposite to the lumen) due to surgical cleavage of plaque from the intact tunica media. In DS, a much thinner lesion (equivalent to Stary type III) with extensive α -SMA and CD68 cellular positivity is present. Right: From top to bottom. Average values and SD of lesional area (mm^2^), intralesional α-SMA and CD68 stain positivity (% of lesional area) of P and DS segments of all specimens’ sections: a smaller lesional area and a markedly higher positivity of both cellular markers in the distal side of the plaque is evident (*p<0.05, **p<0.02 paired t-test).

Masson’s trichrome stain, αSMA and CD68 immunostaining representative of P and DS segments are shown in Figure 2; the quantitative analyses for αSMA and CD68 are also reported, demonstrating a significantly higher content of VSMCs and CD68 positive macrophages in the distal side as compared to central plaque region (Figure 2).

### Secretome analysis of endarterectomy specimens

P and DS segments from the 14 enrolled patients were incubated for 24 h in serum free medium, secretomes were collected, protein digested and peptides fractionated by reverse phase chromatography and analyzed by 5600 TripleTOF mass spectrometer.

**Figure 3:**
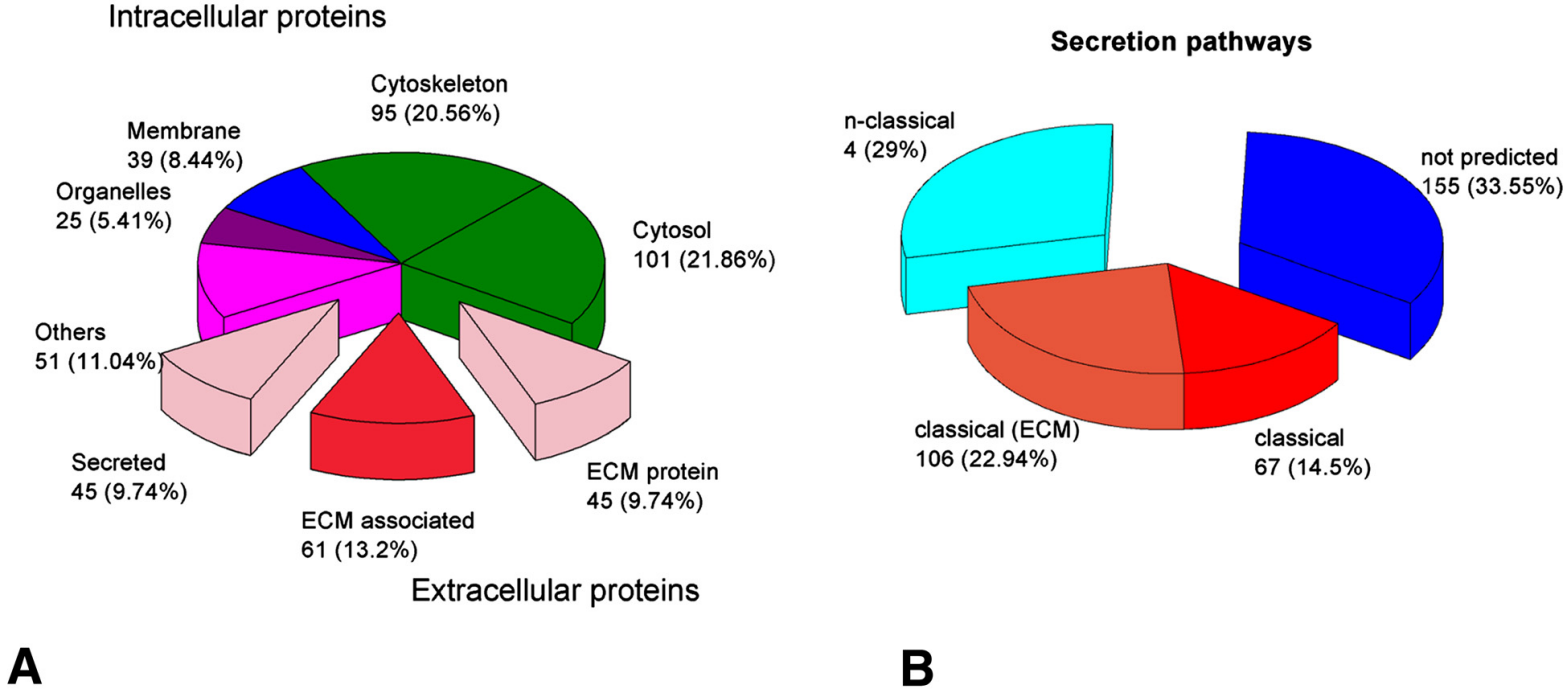
**Pie charts of the total identified proteins from P and DS. A)** Identified proteins are divided on the basis of their localization in intracellular or extracellular space based on Gene Ontology. **B)** Identified proteins are evaluated with SecretomeP software to compute their secretion potential. They were divided in 1) classically secreted 2) not-classically secreted and 3) not-predicted.

Using this LC-MS/MS approach, 463 proteins were identified with a Protein Score (Confidence) > 95% and using local false discovery rate analysis >1% as stringent criterion to avoid false positives (Additional file 1: Table S4 and Additional file 2: Table S5). The entire list of proteins reported in supplementary tables was analyzed in the context of the published literature and firstly grouped as extracellular and intracellular proteins. Using Uniprot database (http://www.uniprot.com), extracellular proteins were further subdivided in ECM proteins and ECM-associated proteins. The intracellular ones were grouped considering their localization in cytosol, membrane, organelle, cytoskeleton and others (Figure 3A).

The secretion potential of these identified proteins was computed by submitting them to SecretomeP server that predicts protein secretion route on the basis of the presence of the signal peptide, responsible for endoplasmic reticulum addressing. The non-classical secretion through multi vesicular bodies was also determined by SecretomeP using specific databanks [21] (Figure 3B).

### Comparative analysis between plaque and its distal counterpart secretomes

In order to evaluate differentially released proteins between P and DS specimens, MarkerView 1.2 software was used and 31 proteins were found differentially secreted. These proteins were classified into two categories: cellular (n=17) and extracellular proteins (n=14) (Table 3). The putative role of each factor in atherosclerosis and cardiovascular disease was reported in the table as suggested by the literature, in order to underline a possible correlation of their dis-regulation to the pathology.

**Table 3:** Differentially released proteins by CEA specimens

**Localization**	**Protein name (1)**	**Gene name**	**Role in atherosclerosis (2)**	**P/DS (3)**	**P value(4)**	**Plasma(5)**
**Cellular**	Alpha-actinin 1	ACTN1	Focal adhesion	▼	6.70E-04	☑
	Integrin-linked protein kinase	ILK	Focal adhesion	▼	1.10E-04	☑
	Actin-related protein 2/3	ARPC3	Focal adhesion	▼	1.20E-04	☑
	Filamin A	FLNA	Focal adhesion	▼	1.35E-05	☑
	PDZ and LIM domain protein 1	PDLI1	Focal adhesion	▼	1.34E-03	☑
	PDZ and LIM domain protein 7	PDLI7	Focal adhesion	▼	5.70E-04	☑
	Vimentin	VIM	Focal adhesion	▼	2.02E-03	☑
	Vinculin	VCL	Focal adhesion [12]	▼	2.80E-04	☑
	Cysteine-rich protein 2	CRIP2	SMC differentiation [22]	▼	1.10E-04	
	Calponin 1	CNN1	SMC differentiation	▼	1.90E-04	
	Calponin 2	CNN2	SMC differentiation	▼	2.57E-03	☑
	Calponin 3	CNN3	SMC differentiation	▼	1.10E-04	
	Transgelin	TAGL	SMC differentiation	▼	3.00E-03	
	Tropomyosin-1 alpha	TPM1	SMC differentiation	▼	2.63E-03	☑
	Tropomyosin beta	TPM2	SMC differentiation	▼	1.14E-03	☑
	Smoothelin	SMOO	SMC differentiation and plaque stability [23]	▲	3.40E-04	☑
	Vascular cell adhesion protein 1 (VCAM-1)	VCAM1	Monocyte recruitment	▼	1.39E-02	☑
**Extracellular matrix (ECM)**	Collagen alpha 1 (III)	COL3A1	Plaque stability	▲	1.70E-04	
	Collagen alpha 1 (VI)	COL6A1	Plaque stability	▲	2.40E-04	
	Collagen alpha 1 (XIV)	COL14A1	Atherogenesis	▲	1.05E-03	
	Collagen alpha 1 (XVI)	COL16A1	Atherogenesis	▲	2.07E-03	
	Versican	VCAN	Atherogenesis [24]	▲	1.45E-05	
	Lumican	LUM	Plaque rupture [24]	▲	4.07E-02	☑
	Fibulin 2	FBLN2	Cell migration [25]	▲	9.00E-03	
	Emilin 1	EMILIN1	Elastic lamellae integrity [26]	▲	1.82E-03	☑
	Periostin	POSTN	Angiogenesis	▲	2.07E-03	
	Thrombospondin-1	THBS1	Angiogenesis [27]	▲	1.71E-02	☑
	Vitamin D binding protein	GC	Acute events [28,29]	▲	1.93E-02	☑
	Apolipoprotein A-II	APOA2	Lipid transport	▲	1.28E-03	☑
	Apolipoprotein D	APOD	Lipid transport	▲	3.11E-05	☑
	Aggrecan	ACAN	Lipoprotein retention	▲	1.51E-05	

(1) Protein name is reported according with SwissProt 2011 database. (2) Role in atherosclerosis is referred to data reported on the recent literature (3) ▼: down released in P compared with DS; ▲: up released in P vs DS. (4) P values were generated using Marker View software. (5) Their presence in plasma was showed according to Human Protein Reference database (http://www.hprd.org).

**Figure 4:**
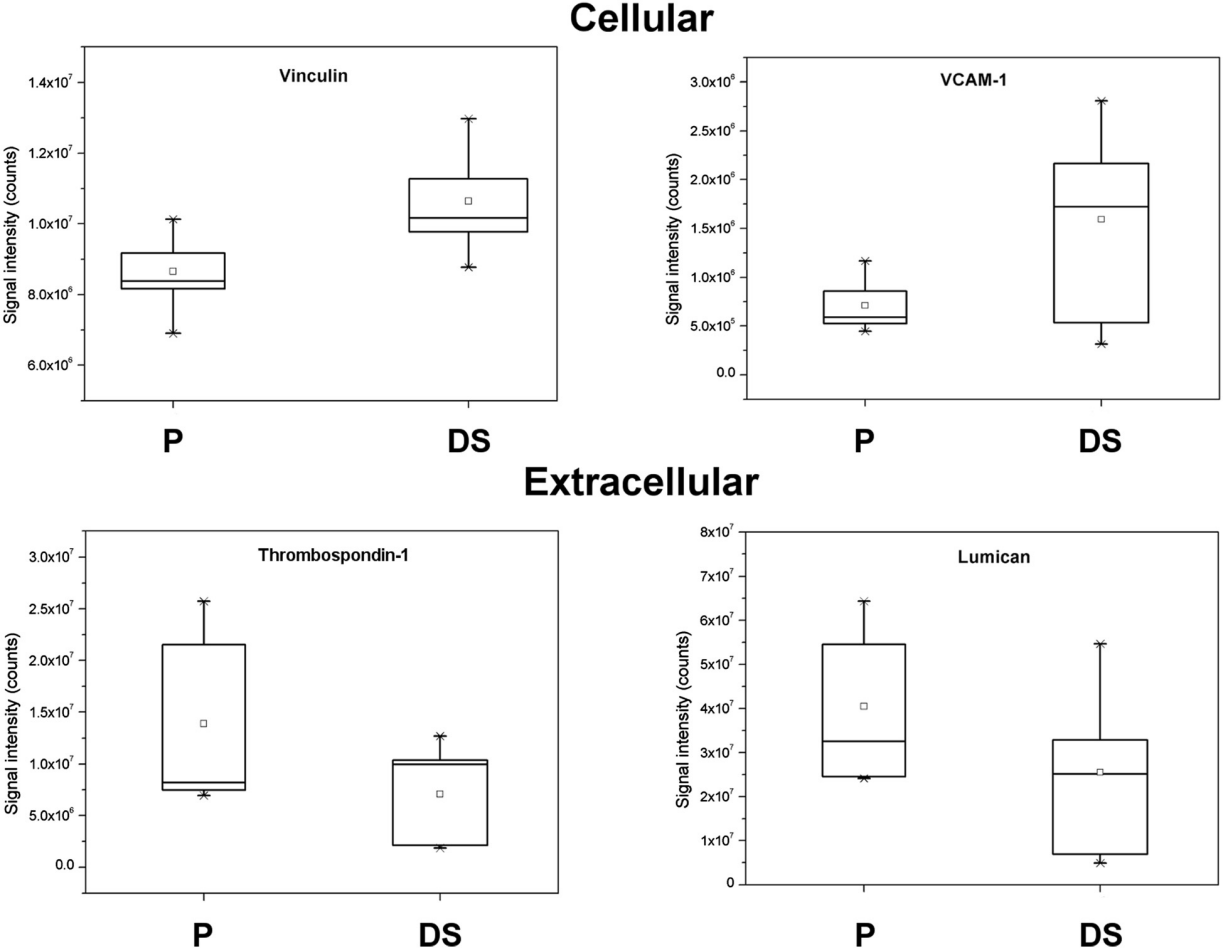
**Differential protein expression obtained by mass spectrometric analysis of P and DS.** Four of the 31 proteins reported in table 3 are chosen and comparative expression analysis is presented as Box plots. Box plots show median value, standard deviation (SD), 50%, minimum and maximum intensity values and were exported from Marker View software.

Nine out of the 14 extracellular proteins were found over-expressed in P secretome, whilst cellular proteins were found over-expressed in DS secretome, with the exception of smoothelin, which is a recognized marker of VSMC contractile phenotype [23]. Of these proteins eighteen have been already reported in serum/plasma and listed in Human Protein Reference database (http://www.hprd.org). Origin 7.0 was used to represent the modulation of the differentially released proteins and the most interesting are reported as representatives of the results (Figure 4).

### Validation by Western Blot and ELISA assay

**Figure 5:**
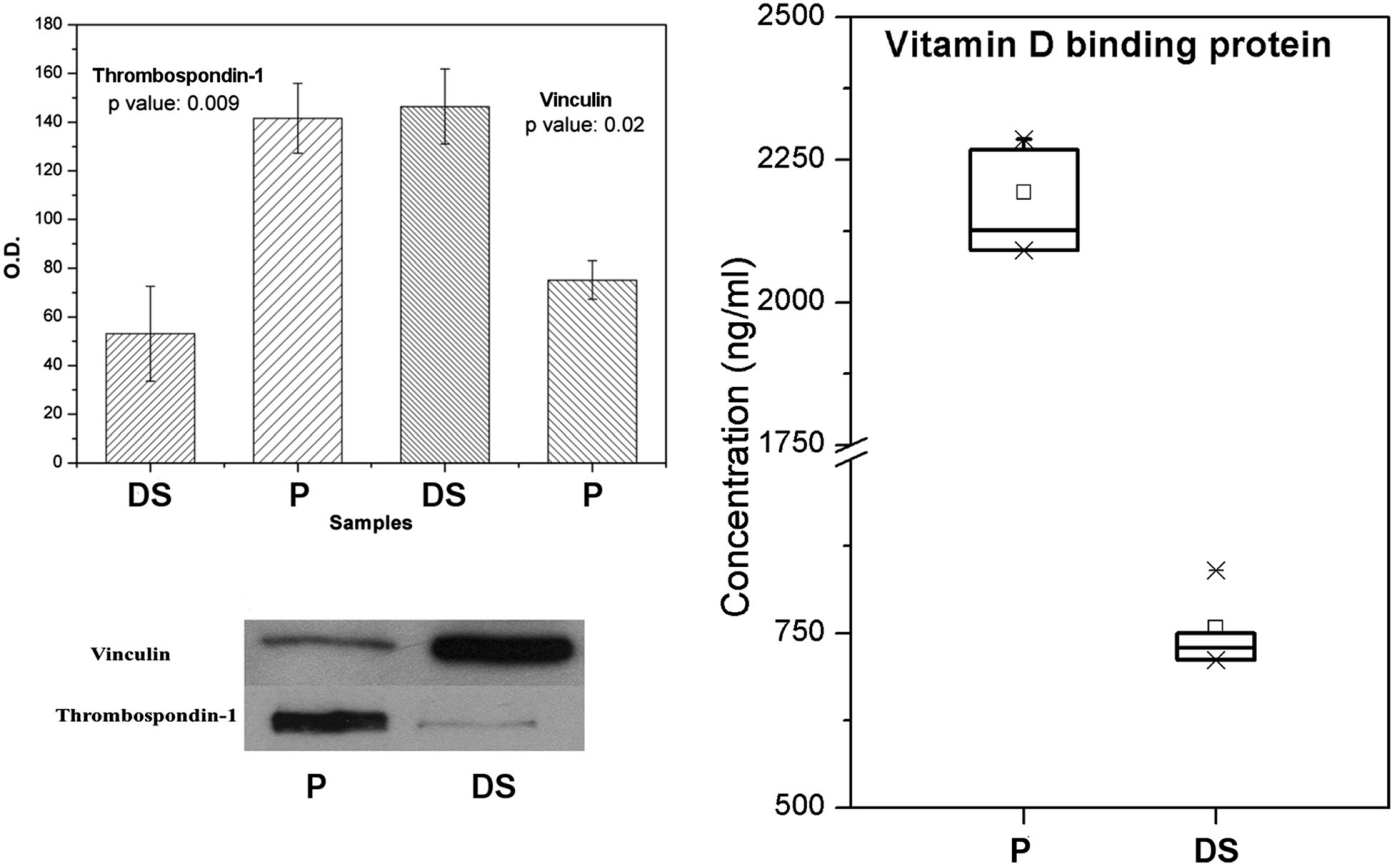
**Left: Western blot analysis of P and DS secretomes.** Densitometric quantification of vinculin and thrombospondin-1 using Quantity One 1D analysis software. Representative images: 10 μg of proteins were separated on SDS-PAGE and blotted on nitrocellulose membrane. Immunoblots were probed with antibodies against vinculin and thrombospondin-1. Right: ELISA assay for vitamin D binding protein expression in secretome samples (P and DS).

Western Blot analysis and also ELISA assay were applied to secretomes in order to validate the differential release observed by mass spectrometric analysis. Vinculin and thrombospondin-1 were selected for Western blot validation (Figure 5A), while vitamin D binding protein was selected for ELISA assay (Figure 5B).

### Immunohistochemistry quantification

**Figure 6:**
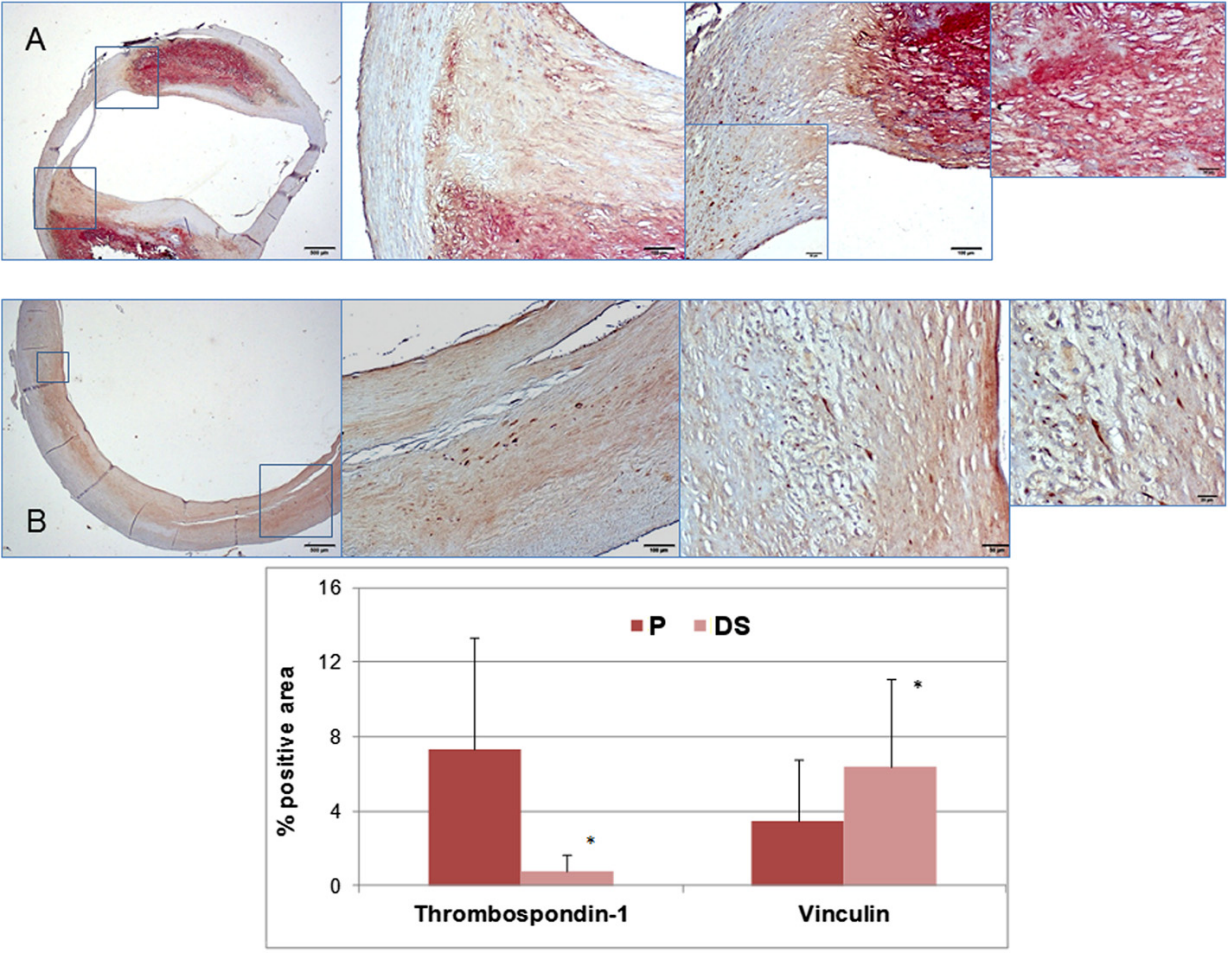
**Top: Double immunostaining of thrombospondin-1 (red) and vinculin (brown) in P (A) and DS (B) sections of a fibrocalcific type Vb plaque.** Low magnification (2×, left) and high magnification (10× to 40×) microscopic fields (insets) are shown. Neither cellular nor extracellular co-distribution of the two antibodies is present. A selective cellular binding of vinculin both on P an DS sections is evident, while thrombospondin-1 is almost exclusively located in the fibrocalcific core of P. Bottom: Average values and SD of thrombospondin-1 and vinculin positivity (single immunostaining) in lesional area of all CEA specimens showing a markedly greater stain in P as compared toDS for thrombospondin-1 and an opposite pattern for vinculin. *p<0.02 paired t-test.

Double immunostaining of anti-vinculin and anti-thrombospondin-1 antibodies on P and DS sections of a CEA specimen is shown in Figure 6. Anti-vinculin antibody selectively stains lesional cells whereas thrombospondin-1 stain is confined to the plaque lipid-necrotic core and surrounding fibrous cap.

Quantitative results derived from single immunostaining of both markers on sections of all CEA specimens confirm the significantly higher (6.4±5.0 vs 3.4±3.0 P<0.02) vinculin positivity and the much lower (0.8±0.8 vs 7.3±6.0 P<0.02) thrombospondin-1 positivity in the lesion area of DS as compared to corresponding P segments.

### ELISA assay of plasma samples

**Figure 7:**
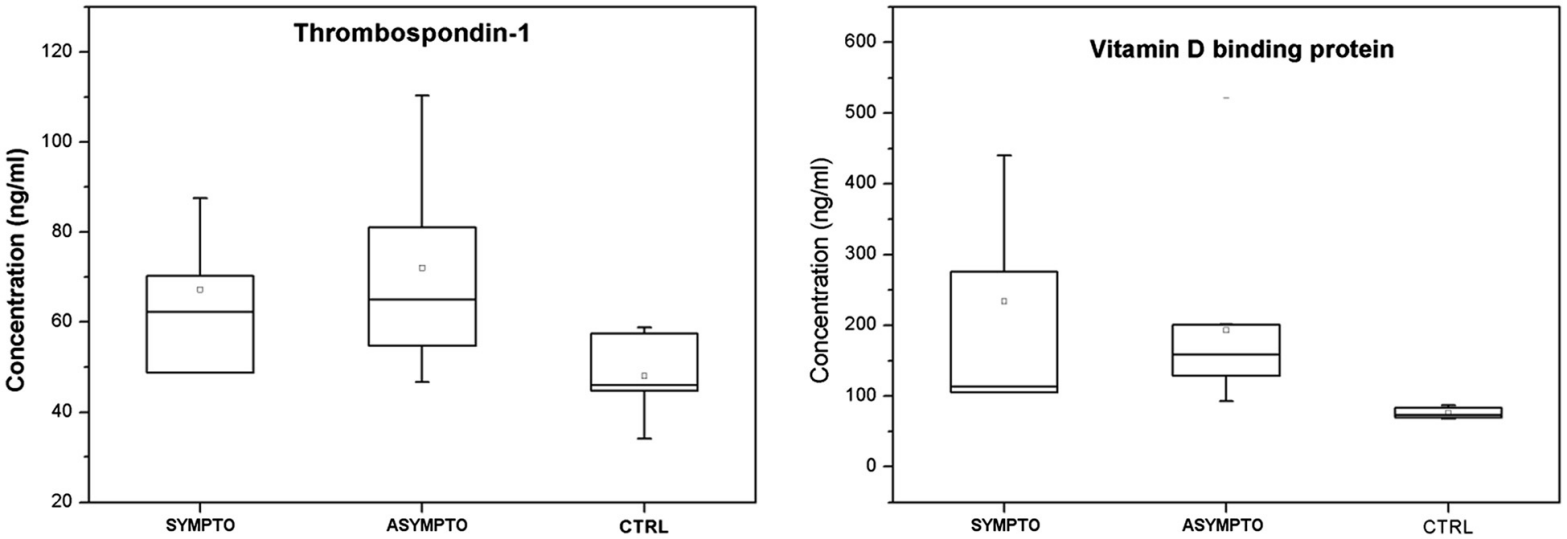
ELISA assay for thrombospondin-1 and vitamin D binding protein expression in plasma samples of 34 CEA patients classified as symptomatic and asymptomatic and 10 healthy controls.

Dosage by double-antibody sandwich ELISA assays of thrombospondin-1 and vitamin D binding protein were performed in plasma samples of 34 CEA patients and 10 age-matched healthy controls. As reported in Table 1, 11 patients were symptomatic while 23 were asymptomatic. Plasma levels of thrombospondin-1 and vitamin D binding protein were significantly higher in atherosclerotic patients than in healthy subjects (thrombospondin-1: 71±20 vs 48±9 ng/mL P value= 0.02; Vitamin D binding protein: 205±133 vs 76±8 ng/mL P value= 0.05), while no significant differences were observed between symptomatic and asymptomatic patients (thrombospondin-1: 67±14 vs 72±20 ng/mL P value=0.72; vitamin D binding protein: 234±137 vs 192±122 ng/mL P value=0.69) (Figure 7).

## Discussion

Several proteomics approaches have been carried out on in order to clarify the mechanisms of atherogenesis as well as to search for plaque presence and severity. The majority of these studies concern carotid atherosclerosis.

All these studies, due to technical limits as well as to low sensitive and low-throughput methods, identified only a small number of proteins, restricting considerably the detection of novel low abundant biomarkers.

Lepedda et al. [6] identified 9 differentially expressed proteins in unstable compared to stable plaques while, only one year later, Olson et al. [7] suggested 19 proteins with a differential distribution in stable compared to more complicated carotid segments of the same patients. To increase sensitivity in the search of new biomarkers, high-throughput western blot analyses and microarray technology were performed allowing the finding of 7 differentially expressed proteins [8] and 11 proteins distributed between stable and unstable plaques [30]. A tissue proteomics study has recently identified and demonstrated that osteopontin tissue expression in carotid plaques and its plasma levels are predictive of adverse cardiovascular events in all atherosclerotic vascular districts [9].

One of the first pioneering secretome studies, using two-dimensional gel electrophoresis, allowed the identification of 14 proteins secreted by autoptic human coronary artery segments [12], but only recently, in order to avoid high abundant plasma protein contamination, Cooksley-Decasper and colleagues [13] studied the secretomes from carotid plaques combining antibody phage display with mass spectrometry. This last approach couples sensitivity and accuracy providing a direct detection of secreted proteins in blood. The method is quite complex respect to the full-screen untargeted approach chosen in our study and the number of identified proteins is rather limited, thus restricting the “discovery potential” of that approach.

In our study the secretome of human carotid plaques was tackled with a high performance, very sensitive, gel- and label-free LC-MS/MS workflow. The unbiased approach, able to give a high sensitive full screen overview of secreted proteins from human carotid artery, is presented here for the first time. This method allowed the identification of 463 proteins thus producing a wide shot on proteins released by atherosclerotic arteries. The majority of the identified proteins (70%) are predicted as secretory proteins by SecretomeP software. 40% of these, due to the presence of the characteristic signal peptide, are likely to follow the classical exocytosis pathway across the Golgi apparatus. The other proteins are non-classically secreted through microvesicles and multi vesicular bodies-derived exosomes, as evidenced by the inclusion in ExoCarta databank (http://www.exocarta.org).

In our procedure, CEA specimens were cut in P and downstream DS segments in order to compare the secretomes of the upstream area of maximum stenosis to that of partially preserved downstream area, subjected to distinct flow shear stress and wall strain regimen. P segments were classified as Stary’s types V and VI [19,20], while DS segments displayed less severe wall changes. We considered this approach useful to differentiate specific molecular factors of the central plaque area, where complication features are present, from those of its distal side.

Indeed, while the majority of the hitherto performed studies have been focused on plaque classification and are based on cross-sectional observations, without taking into account the longitudinal heterogeneity and asymmetry within the atherosclerotic CEA specimens, it has been recently recognized that the upstream side is associated with an increased incidence of severe lesions with cap rupture, while the downstream side is associated with higher VSMC content and it could thus be more representative of cellular growth [14,17,18].

According to this line of reasoning, we found that proteins involved in ECM organization are more represented in P secretome while proteins concerning VSMC phenotype switch, cell contraction and migration, as well as focal adhesion pathways are more abundantly released by DS segments. These observations confirm and strengthen the role of VSMC activation in the early stages of plaque development [31,32] and, conversely, the role of ECM substrates and matrix remodeling in mature plaques [24,26].

**Figure 8:**
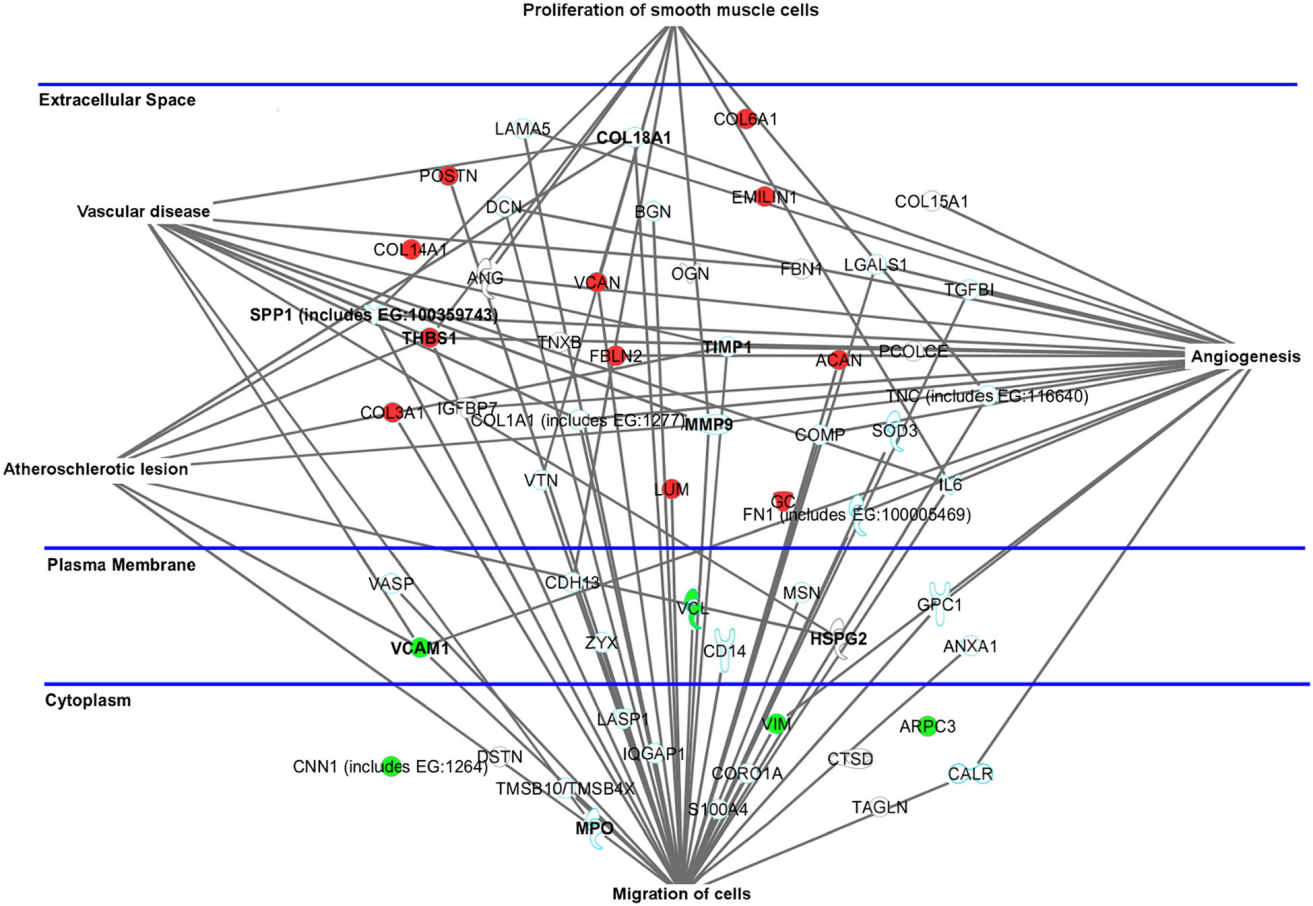
**Ingenuity Pathway Analysis (IPA) of a subset of 56 proteins identified in the secretome of CEA specimens.** Pathways were generated based on the information stored in IPA Knowledge base. Nodes correspond to the 56 proteins and are reported with their Gene Codes. Colors correspond to differentially released proteins between P and DS reported in Table 3. Red: up-released by P. Green: down-released by P. Extracellular space, cytoplasm and plasma membrane proteins are shown based on IPA classification.

Pathway analysis was chosen to relate identified proteins to their cell function and/or pathological role in vascular diseases. As evidenced by IPA clusters (Figure 8) almost all proteins reported as involved in atherosclerotic lesion and vascular disease are also related to migration and proliferation of cells, reinforcing the primary role of VSMCs in plaque progression.

Secretome expression of vinculin and thrombospondin-1 have been validated by western blot analyses: the first one being an intracellular protein, involved in cell migration, the latter one as representative of ECM-related proteins and reported to control atherosclerotic plaque maturation [12,27]. Single and double immunostaining confirmed western blot results of differential protein distribution along CEA specimens, at the same time excluding significant tissue colocalization. A sandwich ELISA assay was also used to confirm the differential secretion of vitamin D binding protein, an extracellular protein reported as marker of acute events [28,29]. Both western blots and ELISA tests validated the mass data on secretomes.

Amongst the 463 identified proteins, 31 resulted differentially secreted by P and DS samples. Half of them were extracellular factors, most of which already reported in plasma, according to Human Protein Reference Database, suggesting that, once released from tissue, they could be found in peripheral blood. For this reason, vitamin D binding protein and thrombospondin-1 were measured in plasma samples of CEA candidates and found significantly higher than in non atherosclerotic subjects. No significant association with symptomatology was evidenced. The correlation between secretome modulation and plasma levels of these two proteins confirms that tissue-secreted protein analysis can be helpful to search for novel disease-specific biomarkers in blood and improve non-invasive risk assessment.

The clinical impact of secretome approach to vascular pathology and atherosclerosis arises from the potential use of plaque-secreted molecules as useful predictors of disease outcome and/or of risk assessment. An untargeted secretome strategy can be able to differentiate progressing from stable plaques as well as discriminating rupture-prone from non-vulnerable plaques, leading to a proper selection and optimal management of high risk patients [10].

This potentiality is challenged by clinical and biological limitations: (a) atherothrombosis accounts for no more than 20% of strokes; (b) complicated ruptured plaques can be asymptomatic, limiting markers specificity; (c) site-specificity of arterial VSMCs and extent of plaque vascularization can influence type and quantity of proteins released in the blood.

Despite these restrictions, secretome analysis seems a most valuable tool to disclose the molecular basis of atherosclerosis as well as of several other diseases [33], due to the unique combination of tissue specificity of biomarkers and clinical feasibility of their detection in blood, aimed to improve patient diagnosis and monitoring.

Results of an untargeted secretome analysis are presented in this study: this unbiased strategy, as compared to targeted analysis, may complicate a prompt clinical output but it is surely the most adequate approach to unravel novel disease markers.

The relatively small number of CEA specimens analyzed represents a limit and the lack of sample stratification is the most relevant consequence of this fact. Despite that, our high sensitive method evidences a very broad spectrum of proteins in carotid plaque secretome and comparative analysis between P and its DS overrides inter-patient variability.

Two ECM proteins released by P are found significantly higher in plasma from atherosclerotic patients without differences between symptomatic and asymptomatic ones, supporting the mismatch between plaque-related and event-related factors, in agreement with other findings [9].

A further clinical relevance of the present study relies on the evidence that a large number of CEA secreted proteins are asymmetrically distributed along the atherosclerotic vessel, related to plaque complication features: this implies that a broader cellular and extracellular protein profiling, rather than isolated marker assays, may be required to increase specificity and predictive potentiality. Future studies are needed to specify secretome-plasma proteins profiling at different stages of carotid plaque growth and complication and its relation to clinical events.

## Conclusions

Thanks to an optimized workflow, a detailed, broad spectrum profile of the human carotid plaque secreted proteins has been obtained. A large number of CEA-secreted proteins are evidenced which result asymmetrically distributed along the atherosclerotic vessel according to their cellular or extracellular origin, suggesting that markers of stable plaque growth and those of plaque complication could be exploited as biomarkers of distinct and specific stages of disease.

## Competing interests

The authors declare that they have no competing interests.

## Authors’ contributions

SRoc, AC and MGT conceived and designed the study. MF and MM performed CEA and collected clinical data. AC set up CEA secretomes. SRoc and SRos performed LC-MS/MS workflow. SRoc and GP analyzed data. SRos and AC performed western blots and ELISA assays. FV and GP performed histology and immunohistology. GP and SRoc wrote the manuscript. LC, MGT, GP and SRoc revised the manuscript. All authors read and approved the final manuscript.

## Supplementary Material

Additional file 1: Table S4 Cellular identified proteins using LC- MS/MS approach. Click here for file

Additional file 2: Table S5 Extra cellular identified proteins using LC- MS/MS approach. Click here for file
