# Supplementary material for: Secreted proteins from carotid endarterectomy: an untargeted approach to disclose molecular clues of plaque progression
Source: J Transl Med. 2013 Oct 16;11:260. doi: 10.1186/1479-5876-11-260 (PMC3853772; doi:10.1186/1479-5876-11-260)
Supplement: Additional file 1: Table S4 — Cellular identified proteins using LC- MS/MS approach. [file 1479-5876-11-260-S1.docx]

| **Paragon Score** | **%Cov(95)** | **Accession** | **Entry Name** | **Name** | **Peptides(95%)** | **SecretomeP** | **localization** |
| --- | --- | --- | --- | --- | --- | --- | --- |
| **CYTOSOL** |  |  |  |  |  |  |  |
| 19,41 | 52,0 | P31946 | 1433B | 14-3-3 protein beta/alpha | 15 | -- | cytoplasm |
| 11,4 | 47,1 | P62258 | 1433E | 14-3-3 protein epsilon | 10 | -- | cytoplasm |
| 8,21 | 38,5 | P61981 | 1433G | 14-3-3 protein gamma | 11 | -- | cytoplasm |
| 11,88 | 40,4 | P27348 | 1433T | 14-3-3 protein theta | 9 | -- | cytoplasm |
| 27,7 | 62,0 | P63104 | 1433Z | 14-3-3 protein zeta/delta | 20 | -- | cytoplasm |
| 4,43 | 8,3 | P52209 | 6PGD | 6-phosphogluconate dehydrogenase | 2 | -- | Cytoplasm |
| 2,28 | 10,5 | Q96IU4 | AB14B | Abhydrolase domain-containing protein 14B | 1 | -- | Cytoplasm |
| 2,02 | 2,0 | P49588 | SYAC | Alanyl-tRNA synthetase, cytoplasmic | 1 | -- | Cytoplasm |
| 44,34 | 73,6 | P00325 | ADH1B | Alcohol dehydrogenase 1B | 26 | -- | Cytoplasm |
| 5,46 | 6,1 | P14619 | KGP1B | cGMP-dependent protein kinase 1, beta isozyme | 3 | -- | Cytoplasm |
| 9,35 | 32,8 | P12277 | KCRB | Creatine kinase B-type | 7 | -- | Cytoplasm |
| 3,67 | 11,2 | P28838 | AMPL | Cytosol aminopeptidase | 4 | -- | Cytoplasm |
| 7,68 | 21,5 | Q96KP4 | CNDP2 | Cytosolic nonspecific dipeptidase | 7 | -- | Cytoplasm |
| 10,73 | 39,9 | Q16555 | DPYL2 | Dihydropyrimidinase-related protein 2 | 13 | -- | cytoplasm |
| 8,71 | 22,5 | P68104 | EF1A1 | Elongation factor 1-alpha 1 | 7 | -- | cytoplasm |
| 12,07 | 16,0 | P13639 | EF2 | Elongation factor 2 | 10 | -- | cytoplasm |
| 3,27 | 13,5 | P05413 | FABPH | Fatty acid-binding protein | 3 | -- | cytoplasm |
| 8,06 | 59,3 | Q01469 | FABPE | Fatty acid-binding protein, epidermal | 6 | -- | cytoplasm |
| 17,31 | 31,7 | P06744 | G6PI | Glucose-6-phosphate isomerase | 10 | -- | Cytoplasm |
| 52,46 | 76,4 | P04406 | G3P | Glyceraldehyde-3-phosphate dehydrogenase | 44 | -- | Cytoplasm |
| 42,07 | 46,8 | P08107 | HSP71 | Heat shock 70 kDa protein 1 | 28 | -- | cytoplasm |
| 30,16 | 37,1 | P11142 | HSP7C | Heat shock cognate 71 kDa protein | 22 | -- | cytoplasm |
| 24,14 | 27,3 | P07900 | HS90A | Heat shock protein HSP 90-alpha | 16 | -- | cytoplasm |
| 4,58 | 23,5 | P08238 | HS90B | Heat shock protein HSP 90-beta | 12 | -- | cytoplasm |
| 9,75 | 76,2 | O14558 | HSPB6 | Heat-shock protein beta-6 | 7 | -- | cytoplasm |
| 2,32 | 10,3 | Q16543 | CDC37 | Hsp90 co-chaperone Cdc37 | 2 | -- | cytoplasm |
| 22,52 | 44,9 | Q9NZU5 | LMCD1 | LIM and cysteine-rich domains protein 1 | 14 | -- | cytoplasm |
| 19,99 | 59,8 | P40121 | CAPG | Macrophage capping protein | 15 | -- | cytoplasm |
| 10,22 | 24,5 | P40925 | MDHC | Malate dehydrogenase, cytoplasmic | 6 | -- | Cytoplasm |
| 2 | 14,4 | P58546 | MTPN | Myotrophin | 1 | -- | cytoplasm |
| 2,26 | 1,4 | P48681 | NEST | Nestin | 2 | -- | cytoplasm |
| 6,45 | 9,5 | Q9BZQ8 | NIBA | Niban protein | 7 | -- | cytoplasm |
| 26,24 | 70,9 | P62937 | PPIA | Peptidyl-prolyl cis-trans isomerase A | 24 | -- | Cytoplasm |
| 14,05 | 26,9 | P36871 | PGM1 | Phosphoglucomutase-1 | 10 | -- | Cytoplasm |
| 67,14 | 61,6 | P13797 | PLST | Plastin-3 | 37 | -- | cytoplasm |
| 2,79 | 10,4 | Q8WUM4 | PDC6I | Programmed cell death 6-interacting protein | 4 | -- | cytoplasm |
| 6,37 | 20,1 | Q06323 | PSME1 | Proteasome activator complex subunit 1 | 3 | -- | cytoplasm |
| 4,37 | 21,3 | Q9UL46 | PSME2 | Proteasome activator complex subunit 2 | 4 | -- | cytoplasm |
| 4,1 | 13,7 | P25786 | PSA1 | Proteasome subunit alpha type 1 | 3 | -- | Cytoplasm |
| 3,22 | 10,2 | P25788 | PSA3 | Proteasome subunit alpha type 3 | 2 | -- | Cytoplasm |
| 5,25 | 17,0 | P28066 | PSA5 | Proteasome subunit alpha type 5 | 3 | -- | Cytoplasm |
| 20,62 | 76,7 | Q99497 | PARK7 | Protein DJ-1 | 14 | -- | cytoplasm |
| 2,24 | 3,0 | P55786 | PSA | Puromycin-sensitive aminopeptidase | 1 | -- | Cytoplasm |
| 3,31 | 22,4 | P31150 | GDIA | Rab GDP dissociation inhibitor alpha | 8 | -- | cytoplasm |
| 15,11 | 26,5 | P50395 | GDIB | Rab GDP dissociation inhibitor beta | 9 | -- | cytoplasm |
| 8,89 | 58,3 | P52565 | GDIR | Rho GDP-dissociation inhibitor 1 | 7 | -- | cytoplasm |
| 8,06 | 35,3 | P52566 | GDIS | Rho GDP-dissociation inhibitor 2 | 5 | -- | cytoplasm |
| 7,7 | 22,1 | Q07960 | RHG01 | Rho GTPase-activating protein 1 | 5 | -- | cytoplasm |
| 2,24 | 1,2 | O75116 | ROCK2 | Rho-associated protein kinase 2 | 1 | -- | cytoplasm |
| 6,6 | 13,8 | Q12765 | SCRN1 | Secernin-1 | 4 | -- | Cytoplasm |
| 2,13 | 3,8 | Q13228 | SBP1 | Selenium-binding protein 1 | 1 | -- | cytoplasm |
| 3,46 | 4,6 | P31948 | STIP1 | Stress-induced-phosphoprotein 1 | 3 | -- | cytoplasm |
| 21,72 | 28,9 | P29401 | TKT | Transketolase | 13 | -- | Cytoplasm |
| 5,33 | 11,7 | P23381 | SYWC | Tryptophanyl-tRNA synthetase, cytoplasmic | 3 | -- | Cytoplasm |
| 2 | 13,1 | Q9H7C9 | CK067 | UPF0366 protein C11orf67 | 1 | -- | cytoplasm |
| 2,01 | 5,6 | Q9P0L0 | VAPA | Vesicle-associated membrane protein | 1 | -- | cytoplasm |
| 2,03 | 4,0 | O60237 | MYPT2 | Protein phosphatase 1 regulatory subunit 12B | 3 | -- | Cytoplasm |
| 3,54 | 8,0 | P11021 | GRP78 | 78 kDa glucose-regulated protein precursor | 5 | classical | cytoplasm |
| 2,79 | 4,5 | Q8N436 | CPXM2 | Carboxypeptidase-like protein X2 | 2 | classical | Cytoplasm |
| 2,16 | 2,4 | P14625 | ENPL | Endoplasmin precursor | 1 | classical | cytoplasm |
| 2,07 | 7,1 | P03952 | KLKB1 | Plasma kallikrein | 3 | classical | Cytoplasm |
| 22,26 | 32,2 | P07602 | SAP | Proactivator polypeptide | 23 | classical | Cytoplasm |
| 8,41 | 18,7 | P07237 | PDIA1 | Protein disulfide-isomerase | 6 | classical | Cytoplasm |
| 2,14 | 7,2 | Q5JXB2 | UE2NL | Putative ubiquitin-conjugating enzyme E2 N-like | 1 | classical | Cytoplasm |
| 2 | 7,0 | P46952 | 3HAO | 3-hydroxyanthranilate 3,4-dioxygenase | 1 | non-classical | Cytoplasm |
| 3,72 | 31,1 | P25398 | RS12 | 40S ribosomal protein S12 | 3 | non-classical | cytoplasm |
| 4 | 20,9 | O95336 | 6PGL | 6-phosphogluconolactonase | 2 | non-classical | Cytoplasm |
| 2,47 | 17,5 | P00568 | KAD1 | Adenylate kinase isoenzyme 1 | 2 | non-classical | Cytoplasm |
| 2,15 | 3,3 | O43488 | ARK72 | Aflatoxin B1 aldehyde reductase member 2 | 1 | non-classical | Cytoplasm |
| 4,44 | 15,1 | P14550 | AK1A1 | Alcohol dehydrogenase [NADP+] | 4 | non-classical | Cytoplasm |
| 65,9 | 77,0 | P06733 | ENOA | Alpha-enolase | 44 | non-classical | Cytoplasm |
| 7,11 | 36,9 | Q15121 | PEA15 | Astrocytic phosphoprotein PEA-15 | 4 | non-classical | cytoplasm |
| 3,61 | 8,5 | P00918 | CAH2 | Carbonic anhydrase 2 | 2 | non-classical | Cytoplasm |
| 5,66 | 15,5 | P16152 | DHCA | Carbonyl reductase [NADPH] 1 | 3 | non-classical | Cytoplasm |
| 8,29 | 70,4 | P04080 | CYTB | Cystatin-B | 5 | non-classical | Cytoplasm |
| 25,63 | 40,6 | Q13642 | FHL1 | Four and a half LIM domains protein 1 | 17 | non-classical | cytoplasm |
| 2,07 | 14,7 | Q14192 | FHL2 | Four and a half LIM domains protein 2 | 3 | non-classical | cytoplasm |
| 6,35 | 18,8 | P17931 | LEG3 | Galectin-3 | 4 | non-classical | Cytoplasm |
| 3,38 | 8,6 | P46976 | GLYG | Glycogenin-1 | 2 | non-classical | cytoplasm |
| 31,51 | 87,3 | P04792 | HSPB1 | Heat-shock protein beta-1 | 25 | non-classical | cytoplasm |
| 7,11 | 10,0 | P50502 | F10A1 | Hsc70-interacting protein | 4 | non-classical | cytoplasm |
| 46,63 | 84,9 | P00338 | LDHA | L-lactate dehydrogenase A chain | 30 | non-classical | Cytoplasm |
| 14,5 | 32,6 | P07195 | LDHB | L-lactate dehydrogenase B chain | 11 | non-classical | Cytoplasm |
| 4,31 | 17,4 | P14174 | MIF | Macrophage migration inhibitory factor | 3 | non-classical | Cytoplasm |
| 8,49 | 49,5 | O95865 | DDAH2 | NG,NG-dimethylarginine dimethylaminohydrolase 2 | 7 | non-classical | Cytoplasm |
| 5,9 | 32,7 | P30044 | PRDX5 | Peroxiredoxin-5, mitochondrial | 3 | non-classical | Cytoplasm |
| 18,52 | 75,4 | P30086 | PEBP1 | Phosphatidylethanolamine-binding protein 1 | 12 | non-classical | Cytoplasm |
| 3,23 | 7,1 | Q96IZ0 | PAWR | PRKC apoptosis WT1 regulator protein | 2 | non-classical | cytoplasm |
| 2,79 | 3,1 | O14974 | MYPT1 | Protein phosphatase 1 regulatory subunit 12A | 2 | non-classical | Cytoplasm |
| 2,11 | 10,9 | Q96A00 | PP14A | Protein phosphatase 1 regulatory subunit 14A | 1 | non-classical | Cytoplasm |
| 5,66 | 16,4 | P00352 | AL1A1 | Retinal dehydrogenase 1 | 6 | non-classical | Cytoplasm |
| 31,97 | 62,5 | P13489 | RINI | Ribonuclease inhibitor | 19 | non-classical | Cytoplasm |
| 2,64 | 6,6 | P30153 | 2AAA | Serine/threonine-protein phosphatase 2A | 2 | non-classical | Cytoplasm |
| 4,14 | 24,8 | P10768 | ESTD | S-formylglutathione hydrolase | 5 | non-classical | Cytoplasm |
| 12,9 | 84,2 | O75368 | SH3L1 | SH3 domain-binding glutamic acid-rich-like protein | 8 | non-classical | cytoplasm |
| 5,74 | 67,7 | Q9H299 | SH3L3 | SH3 domain-binding glutamic acid-rich-like protein 3 | 6 | non-classical | cytoplasm |
| 5,03 | 11,2 | Q99536 | VAT1 | Synaptic vesicle membrane protein VAT-1 homolog | 3 | non-classical | Cytoplasm |
| 2,14 | 7,0 | Q16881 | TRXR1 | Thioredoxin reductase 1, cytoplasmic precursor | 2 | non-classical | Cytoplasm |
| 6,65 | 51,7 | P13693 | TCTP | Translationally-controlled tumor protein | 5 | non-classical | cytoplasm |
| 9,16 | 12,0 | P22314 | UBE1 | Ubiquitin-activating enzyme E1 | 8 | non-classical | Cytoplasm |
| 2,23 | 4,3 | P21281 | VATB2 | Vacuolar ATP synthase subunit B, brain isoform | 1 | non-classical | cytoplasm |
| **CYTOSKELETON** | |  |  |  |  |  |  |
| 5,68 | 14,2 | P61160 | ARP2 | Actin-like protein 2 | 4 | -- | cytoskeleton |
| 14,93 | 39,2 | P61158 | ARP3 | Actin-like protein 3 | 10 | -- | cytoskeleton |
| 5,44 | 13,7 | O15143 | ARC1B | Actin-related protein 2/3 complex subunit 1B | 4 | -- | cytoskeleton |
| 3,67 | 20,7 | O15144 | ARPC2 | Actin-related protein 2/3 complex subunit 2 | 4 | -- | cytoskeleton |
| 2,24 | 1,2 | Q02952 | AKA12 | A-kinase anchor protein 12 | 1 | -- | cytoskeleton |
| 155,29 | 78,8 | P12814 | ACTN1 | Alpha-actinin-1 | 110 | -- | cytoskeleton |
| 134,94 | 57,8 | Q05682 | CALD1 | Caldesmon | 91 | -- | cytoscheleton |
| 2,02 | 3,3 | Q13409 | DC1I2 | Cytoplasmic dynein 1 intermediate chain 2 | 1 | -- | cytoskeleton |
| 7,3 | 5,4 | O15061 | DMN | Desmuslin | 6 | -- | cytoskeleton |
| 36,72 | 54,9 | Q14195 | DPYL3 | Dihydropyrimidinase-related protein 3 | 22 | -- | cytoskeleton |
| 2,57 | 5,2 | Q16643 | DREB | Drebrin | 2 | -- | cytoskeleton |
| 3,49 | 8,7 | Q13561 | DCTN2 | Dynactin subunit 2 | 2 | -- | cytoskeleton |
| 2,02 | 0,7 | P11532 | DMD | Dystrophin | 1 | -- | cytoskeleton |
| 4,24 | 20,3 | P52907 | CAZA1 | F-actin capping protein subunit alpha-1 | 4 | -- | cytoskeleton |
| 6,51 | 19,2 | P47755 | CAZA2 | F-actin capping protein subunit alpha-2 | 5 | -- | cytoskeleton |
| 2,5 | 6,5 | Q16658 | FSCN1 | Fascin | 2 | -- | cytoscheleton |
| 403,02 | 75,7 | P21333 | FLNA | Filamin-A | 352 | -- | cytoskeleton |
| 3,11 | 6,2 | O75369 | FLNB | Filamin-B | 16 | -- | cytoskeleton |
| 8,4 | 7,5 | Q32MZ4 | LRRF1 | Leucine-rich repeat flightless-interacting protein 1 | 5 | -- | cytoskeleton |
| 7,17 | 14,5 | P27816 | MAP4 | Microtubule-associated protein 4 | 7 | -- | cytoskeleton |
| 4,76 | 16,0 | Q15691 | MARE1 | Microtubule-associated protein RP/EB | 3 | -- | cytoskeleton |
| 29,56 | 12,2 | Q15746 | MYLK | Myosin light chain kinase | 23 | -- | cytoskeleton |
| 13,51 | 61,6 | P60660 | MYL6 | Myosin light polypeptide 6 | 13 | -- | cytoskeleton |
| 3,05 | 4,8 | Q6WCQ1 | MRIP | Myosin phosphatase Rho-interacting protein | 3 | -- | cytoskeleton |
| 68,71 | 21,2 | P35580 | MYH10 | Myosin-10 | 48 | -- | cytoskeleton |
| 86,11 | 32,5 | P35749 | MYH11 | Myosin-11 | 59 | -- | cytoskeleton |
| 134,54 | 37,7 | P35579 | MYH9 | Myosin-9 | 86 | -- | cytoskeleton |
| 20,58 | 66,0 | O00151 | PDLI1 | PDZ and LIM domain protein 1 | 14 | -- | cytoskeleton |
| 7,82 | 28,2 | P50479 | PDLI4 | PDZ and LIM domain protein 4 | 6 | -- | cytoskeleton |
| 19,31 | 37,9 | Q96AC1 | PKHC1 | Pleckstrin homology domain | 12 | -- | cytoskeleton |
| 35,82 | 9,1 | Q15149 | PLEC1 | Plectin-1 | 32 | -- | cytoskeleton |
| 23,54 | 87,1 | P07737 | PROF1 | Profilin-1 | 21 | -- | cytoskeleton |
| 2,23 | 10,2 | Q15019 | SEPT2 | Septin-2 | 2 | -- | cytoskeleton |
| 6,21 | 8,0 | P53814 | SMOO | Smoothelin | 4 | -- | cytoskeleton |
| 12,79 | 6,0 | Q13813 | SPTA2 | Spectrin alpha chain, brain | 9 | -- | cytoskeleton |
| 7,8 | 3,3 | Q01082 | SPTB2 | Spectrin beta chain, brain 1 | 5 | -- | cytoskeleton |
| 4,03 | 4,0 | Q8N3V7 | SYNPO | Synaptopodin | 3 | -- | cytoskeleton |
| 22,27 | 14,8 | Q9UMS6 | SYNP2 | Synaptopodin-2 | 15 | -- | cytoskeleton |
| 171,5 | 57,0 | Q9Y490 | TLN1 | Talin-1 | 131 | -- | cytoskeleton |
| 2,03 | 5,4 | P48643 | TCPE | T-complex protein 1 subunit epsilon | 1 | -- | cytoskeleton |
| 22,35 | 14,4 | Q9HBL0 | TENS1 | Tensin-1 | 15 | -- | cytoskeleton |
| 10,98 | 21,1 | Q9UGI8 | TES | Testin | 8 | -- | cytoskeleton |
| 51,23 | 91,5 | P67936 | TPM4 | Tropomyosin alpha-4 chain | 107 | -- | cytoskeleton |
| 149,17 | 80,3 | P07951 | TPM2 | Tropomyosin beta chain | 151 | -- | cytoskeleton |
| 19,61 | 44,8 | P68363 | TBAK | Tubulin alpha-ubiquitous chain | 12 | -- | cytoskeleton |
| 95,36 | 86,5 | O43707 | ACTN4 | Alpha-actinin-4 | 115 | -- | cytoskeleton |
| 3,44 | 7,6 | O60504 | VINEX | Vinexin | 3 | -- | cytoskeleton |
| 86,66 | 64,7 | P06396 | GELS | Gelsolin | 59 | classical | cytoskeleton |
| 2,55 | 78,8 | P68032 | ACTC | Actin, alpha cardiac muscle 1 | 174 | non-classical | cytoskeleton |
| 137,54 | 79,0 | P62736 | ACTA | Actin, aortic smooth muscle | 179 | non-classical | cytoskeleton |
| 58,92 | 85,6 | P60709 | ACTB | Actin, cytoplasmic 1 | 170 | non-classical | cytoskeleton |
| 2,26 | 85,6 | P63261 | ACTG | Actin, cytoplasmic 2 | 162 | non-classical | cytoskeleton |
| 2 | 77,9 | P63267 | ACTH | Actin, gamma-enteric smooth muscle | 172 | non-classical | cytoskeleton |
| 7,6 | 27,0 | O15145 | ARPC3 | Actin-related protein 2/3 complex subunit 3 | 4 | non-classical | cytoskeleton |
| 4 | 35,8 | O15511 | ARPC5 | Actin-related protein 2/3 complex subunit 5 | 3 | non-classical | cytoskeleton |
| 15,56 | 32,3 | Q9NVD7 | PARVA | Alpha-parvin | 12 | non-classical | cytoskeleton |
| 8 | 35,6 | P62158 | CALM | Calmodulin | 7 | non-classical | cytoscheleton |
| 63,69 | 82,8 | P51911 | CNN1 | Calponin-1 | 70 | non-classical | cytoskeleton |
| 9,71 | 29,4 | Q99439 | CNN2 | Calponin-2 | 8 | non-classical | cytoskeleton |
| 26,16 | 58,1 | Q15417 | CNN3 | Calponin-3 | 17 | non-classical | cytoskeleton |
| 21,33 | 69,3 | P23528 | COF1 | Cofilin-1 | 18 | non-classical | cytoskeleton |
| 3,67 | 48,2 | Q9Y281 | COF2 | Cofilin-2 | 6 | non-classical | cytoskeleton |
| 4,54 | 8,7 | P31146 | COR1A | Coronin-1A | 2 | non-classical | cytoskeleton |
| 3,93 | 13,3 | Q9BR76 | COR1B | Coronin-1B | 4 | non-classical | cytoskeleton |
| 14,27 | 24,1 | Q9ULV4 | COR1C | Coronin-1C | 10 | non-classical | cytoskeleton |
| 67,42 | 70,4 | P17661 | DESM | Desmin | 64 | non-classical | cytoskeleton |
| 13,7 | 49,7 | P60981 | DEST | Destrin | 12 | non-classical | cytoskeleton |
| 2,26 | 12,5 | Q9NP97 | DLRB1 | Dynein light chain roadblock-type 1 | 1 | non-classical | cytoskeleton |
| 12,94 | 44,8 | P47756 | CAPZB | F-actin capping protein subunit beta | 8 | non-classical | cytoskeleton |
| 9,18 | 41,0 | P02794 | FRIH | Ferritin heavy chain | 7 | non-classical | cytoskeleton |
| 12,36 | 27,6 | Q8WUP2 | FBLI1 | Filamin-binding LIM protein 1 | 9 | non-classical | cytoskeleton |
| 44,35 | 20,2 | Q14315 | FLNC | Filamin-C | 45 | non-classical | cytoskeleton |
| 12,38 | 37,5 | Q14847 | LASP1 | LIM and SH3 domain protein 1 | 9 | non-classical | cytoskeleton |
| 4,31 | 17,4 | P14174 | MIF | Macrophage migration inhibitory factor | 3 | non-classical | cytoskeleton |
| 12,63 | 6,6 | P46821 | MAP1B | Microtubule-associated protein 1B | 9 | non-classical | cytoskeleton |
| 2,02 | 45,0 | P19105 | MLRM | Myosin regulatory light chain 2 | 6 | non-classical | cytoskeleton |
| 21,61 | 66,3 | P24844 | MLRN | Myosin regulatory light chain 2 | 14 | non-classical | cytoskeleton |
| 11,22 | 33,8 | Q53GG5 | PDLI3 | PDZ and LIM domain protein 3 | 8 | non-classical | cytoskeleton |
| 18,3 | 26,9 | Q9NR12 | PDLI7 | PDZ and LIM domain protein 7 | 12 | non-classical | cytoskeleton |
| 48,97 | 71,9 | P13796 | PLSL | Plastin-2 | 35 | non-classical | cytoskeleton |
| 5,25 | 28,7 | P26447 | S10A4 | Protein S100-A4 | 4 | non-classical | cytosckeleton |
| 8,23 | 35,6 | P06703 | S10A6 | Protein S100-A6 | 6 | non-classical | cytosckeleton |
| 2 | 2,6 | Q9UHD8 | SEPT9 | Septin-9 | 1 | non-classical | cytoskeleton |
| 6,43 | 68,2 | P63313 | TYB10 | Thymosin beta-10 | 6 | non-classical | cytoskeleton |
| 35,4 | 100 | P62328 | TYB4 | Thymosin beta-4 | 31 | non-classical | cytoskeleton |
| 38,52 | 90,5 | P37802 | TAGL2 | Transgelin-2 | 54 | non-classical | cytoskeleton |
| 50,6 | 89,6 | P60174 | TPIS | Triosephosphate isomerase | 37 | non-classical | cytoskeleton |
| 6,09 | 62,3 | P06753 | TPM3 | Tropomyosin alpha-3 chain | 67 | non-classical | cytoskeleton |
| 59,86 | 82,7 | P09493 | TPM1 | Tropomyosin-1 alpha chain | 122 | non-classical | cytoskeleton |
| 37,69 | 66,4 | P07437 | TBB5 | Tubulin beta chain | 22 | non-classical | cytoskeleton |
| 5,22 | 62,9 | P68371 | TBB2C | Tubulin beta-2C chain | 20 | non-classical | cytoskeleton |
| 214,04 | 81,3 | P08670 | VIME | Vimentin | 270 | non-classical | cytoskeleton |
| 22,59 | 38,6 | O75083 | WDR1 | WD repeat protein 1 | 13 | non-classical | cytoskeleton |
| 21,46 | 32,7 | Q15942 | ZYX | Zyxin | 21 | non-classical | cytoskeleton |
| **MEMBRANE** |  |  |  |  |  |  |  |
| 32,49 | 51,2 | Q01518 | CAP1 | Adenylyl cyclase-associated protein 1 | 19 | -- | cell membrane |
| 2,32 | 4,4 | P40123 | CAP2 | Adenylyl cyclase-associated protein 2 | 2 | -- | cell membrane |
| 13,03 | 56,8 | P80723 | BASP | Brain acid soluble protein 1 | 7 | -- | cell membrane |
| 14,53 | 25,8 | Q9NZN4 | EHD2 | EH domain-containing protein 2 | 8 | -- | cell membrane |
| 3,24 | 4,9 | P14317 | HCLS1 | Hematopoietic lineage cell-specific protein | 2 | -- | membrane |
| 2,56 | 8,0 | Q13418 | ILK | Integrin-linked protein kinase | 2 | -- | membrane cell projection |
| 41,43 | 57,7 | Q93052 | LPP | Lipoma-preferred partner | 27 | -- | membrane |
| 13,29 | 45,4 | P33241 | LSP1 | Lymphocyte-specific protein 1 | 11 | -- | membrane |
| 68,31 | 78,1 | P14618 | KPYM | Pyruvate kinase isozymes M1/M2 | 42 | -- | Cell membrane |
| 36,48 | 19,0 | Q9BX66 | SRBS1 | Sorbin and SH3 domain-containing protein 1 | 25 | -- | membrane |
| 2 | 2,7 | Q96RF0 | SNX18 | Sorting nexin-18 | 1 | -- | membrane |
| 2,07 | 0,7 | O95425 | SVIL | Supervillin | 1 | -- | membrane cell projection |
| 4 | 9,6 | O15400 | STX7 | Syntaxin-7 | 2 | -- | membrane |
| 3,07 | 2,4 | Q9UDY2 | ZO2 | Tight junction protein ZO-2 | 2 | -- | membrane cell junction |
| 6,86 | 35,9 | P13987 | CD59 | CD59 glycoprotein | 4 | classical | cell membrane |
| 21,25 | 37,8 | P43121 | MUC18 | Cell surface glycoprotein MUC18 | 12 | classical | membrane |
| 5,85 | 6,5 | P50895 | LU | Lutheran blood group glycoprotein | 4 | classical | membrane |
| 14,23 | 41,3 | P08571 | CD14 | Monocyte differentiation antigen CD14 | 9 | classical | cell membrane |
| 6,48 | 10,0 | Q6UX71 | PXDC2 | Plexin domain-containing protein 2 precursor | 4 | classical | membrane |
| 9,89 | 24,3 | P04083 | ANXA1 | Annexin A1 | 6 | non-classical | cell membrane |
| 2,04 | 8,6 | Q96CX2 | KCD12 | BTB/POZ domain-containing protein KCTD12 | 2 | non-classical | cell membrane |
| 4,21 | 7,7 | P07384 | CAN1 | Calpain-1 catalytic subunit | 3 | non-classical | Cell membrane |
| 9,51 | 15,1 | P17655 | CAN2 | Calpain-2 catalytic subunit | 5 | non-classical | Cell membrane |
| 7,11 | 19,8 | P13861 | KAP2 | cAMP-dependent protein kinase type II | 6 | non-classical | Cell membrane |
| 9,1 | 32,9 | P00915 | CAH1 | Carbonic anhydrase 1 | 7 | non-classical | Cell membrane |
| 17,46 | 60,9 | Q9Y696 | CLIC4 | Chloride intracellular channel protein 4 | 12 | non-classical | membrane |
| 7,36 | 4,2 | Q00610 | CLH1 | Clathrin heavy chain 1 | 5 | non-classical | membrane |
| 2,17 | 7,9 | P09497 | CLCB | Clathrin light chain B | 2 | non-classical | membrane |
| 5,46 | 17,2 | P15311 | EZRI | Ezrin | 13 | non-classical | cell membrane |
| 12,1 | 53,2 | P09104 | ENOG | Gamma-enolase | 15 | non-classical | Cell membrane |
| 7,84 | 15,2 | P00338 | IDHC | Isocitrate dehydrogenase [NADP] cytoplasmic | 4 | non-classical | Cell membrane |
| 9,77 | 32,5 | O60664 | M6PBP | Mannose-6-phosphate receptor-binding protein 1 | 8 | non-classical | membrane |
| 72,22 | 55,8 | P26038 | MOES | Moesin | 43 | non-classical | membrane |
| 7,66 | 7,4 | Q06830 | PERM | Myeloperoxidase | 4 | non-classical | membrane |
| 2 | 7,7 | Q9NP98 | MYOZ1 | Myozenin-1 | 1 | non-classical | cell projection |
| 18,03 | 20,5 | Q96HC4 | PDLI5 | PDZ and LIM domain protein 5 | 10 | non-classical | cell junction |
| 2 | 2,4 | O95394 | AGM1 | Phosphoacetylglucosamine mutase | 1 | non-classical | Cell junction |
| 33,58 | 20,1 | P46940 | IQGA1 | Ras GTPase-activating-like protein IQGAP1 | 19 | non-classical | membrane |
| 10,27 | 11,8 | Q86VB7 | C163A | Scavenger receptor cysteine-rich type 1 | 8 | non-classical | membrane |
| 1,08 | 1,8 | P19320 | VCAM1 | Vascular cell adhesion protein 1 | 1 | classical | membrane |
| 115 | 62,8 | P18206 | VINC | Vinculin | 75 | -- | membrane |
| **ORGANELLES** |  |  |  |  |  |  |  |
| 13,44 | 29,6 | P04040 | CATA | Catalase | 9 | -- | Peroxisome |
| 9,14 | 16,6 | P15104 | GLNA | Glutamine synthetase | 5 | -- | Mitochondrion |
| 10,03 | 10,1 | P11216 | PYGB | Glycogen phosphorylase, brain form | 5 | -- | Mitochondrion |
| 2,21 | 6,2 | Q16775 | GLO2 | Hydroxyacylglutathione hydrolase | 1 | -- | Mitochondrion |
| 6,91 | 12,1 | P29536 | LMOD1 | Leiomodin-1 | 5 | -- | Endoplasmic reticulum |
| 5,93 | 37,5 | P22392 | NDKB | Nucleoside diphosphate kinase B | 5 | -- | Melanosome |
| 20,2 | 69,7 | P18669 | PGAM1 | Phosphoglycerate mutase 1 | 16 | -- | Lysosome |
| 3,16 | 11,9 | Q99436 | PSB7 | Proteasome subunit beta type 7 | 2 | -- | Endoplasmic reticulum |
| 2,04 | 10,0 | Q15084 | PDIA6 | Protein disulfide-isomerase A6 | 3 | -- | Mitochondrion |
| 4 | 2,9 | Q9NQC3 | RTN4 | Reticulon-4 | 2 | -- | Endoplasmic reticulum |
| 11,07 | 8,9 | Q9P2E9 | RRBP1 | Ribosome-binding protein 1 | 9 | -- | Endoplasmic reticulum |
| 11,11 | 31,0 | P07858 | CATB | Cathepsin B | 8 | classical | Lysosome Melanosome |
| 23,46 | 44,9 | P07339 | CATD | Cathepsin D | 16 | classical | Lysosome Melanosome |
| 6,07 | 31,8 | P61916 | NPC2 | Epididymal secretory protein E1 | 3 | classical | Endoplasm Lysosome |
| 3,46 | 1,8 | Q86UP2 | KTN1 | Kinectin | 2 | classical | Endoplasmic reticulum |
| 21,19 | 29,7 | P30101 | PDIA3 | Protein disulfide-isomerase A3 | 12 | classical | Endoplasmic reticulum |
| 4,13 | 3,7 | P13667 | PDIA4 | Protein disulfide-isomerase A4 | 2 | classical | Endoplasmic reticulum |
| 20,03 | 68,8 | Q06830 | PRDX1 | Peroxiredoxin-1 | 14 | non-classical | Mitochondrion |
| 8,34 | 52,0 | P32119 | PRDX2 | Peroxiredoxin-2 | 9 | non-classical | Lysosome |
| 21,05 | 39,5 | Q6NZI2 | PTRF | Polymerase I and transcript release factor | 21 | non-classical | Endoplasmic reticulum |
| 2 | 4,6 | P28072 | PSB6 | Proteasome subunit beta type 6 | 1 | non-classical | Endoplasmic reticulum |
| 4,7 | 15,2 | Q92743 | HTRA1 | Serine protease HTRA1 precursor | 4 | non-classical | Vesicle |
| 21,15 | 90,9 | P00441 | SODC | Superoxide dismutase [Cu-Zn] | 13 | non-classical | Endoplasmic reticulum |
| 32,16 | 40,8 | P55072 | TERA | Transitional endoplasmic reticulum ATPase | 23 | non-classical | Endoplasmic reticulum |
| 5,81 | 12,6 | P50552 | VASP | Vasodilator-stimulated phosphoprotein | 4 | non-classical | Endoplasmic reticulum |
| **SECRETED** |  |  |  |  |  |  |  |
| 43,06 | 76,5 | P00558 | PGK1 | Phosphoglycerate kinase 1 | 30 | classical | Secreted |
| 10,94 | 15,9 | P43652 | AFAM | Afamin | 9 | classical | Secreted |
| 30,63 | 50,7 | P02763 | A1AG1 | Alpha-1-acid glycoprotein 1 | 36 | classical | Secreted |
| 10,12 | 49,3 | P19652 | A1AG2 | Alpha-1-acid glycoprotein 2 | 13 | classical | Secreted |
| 18,65 | 32,9 | P01011 | AACT | Alpha-1-antichymotrypsin | 11 | classical | Secreted |
| 40,19 | 54,1 | P04217 | A1BG | Alpha-1B-glycoprotein | 30 | classical | Secreted |
| 3,63 | 6,5 | P08697 | A2AP | Alpha-2-antiplasmin | 2 | classical | Secreted |
| 38,28 | 54,5 | P02765 | FETUA | Alpha-2-HS-glycoprotein | 38 | classical | Secreted |
| 97,36 | 53,2 | P01023 | A2MG | Alpha-2-macroglobulin | 76 | classical | Secreted |
| 6,02 | 31,3 | P03950 | ANGI | Angiogenin | 3 | classical | Secreted |
| 5,48 | 8,9 | P01019 | ANGT | Angiotensinogen | 3 | classical | Secreted |
| 2,14 | 10,3 | P20851 | C4BB | C4b-binding protein beta chain | 2 | classical | Secreted |
| 48,22 | 42,2 | P00450 | CERU | Ceruloplasmin | 34 | classical | Secreted |
| 21,8 | 70,0 | P08294 | SODE | Extracellular superoxide dismutase [Cu-Zn] | 25 | classical | Secreted |
| 2,69 | 2,6 | Q12841 | FSTL1 | Follistatin-related protein 1 | 1 | classical | Secreted |
| 3,91 | 17,7 | P22352 | GPX3 | Glutathione peroxidase 3 | 3 | classical | Secreted |
| 2,02 | 2,7 | P28799 | GRN | Granulins | 1 | classical | Secreted |
| 43,26 | 64,7 | P02790 | HEMO | Hemopexin | 38 | classical | Secreted |
| 23,89 | 39,6 | P04196 | HRG | Histidine-rich glycoprotein | 16 | classical | Secreted |
| 19,37 | 56,7 | Q16270 | IBP7 | Insulin-like growth factor-binding protein 7 | 10 | classical | Secreted |
| 28,69 | 32,4 | P19827 | ITIH1 | Inter-alpha-trypsin inhibitor heavy chain H1 | 19 | classical | Secreted |
| 24,67 | 19,3 | P19823 | ITIH2 | Inter-alpha-trypsin inhibitor heavy chain H2 | 13 | classical | Secreted |
| 2,96 | 5,9 | Q06033 | ITIH3 | Inter-alpha-trypsin inhibitor heavy chain H3 | 3 | classical | Secreted |
| 28,26 | 31,6 | Q14624 | ITIH4 | Inter-alpha-trypsin inhibitor heavy chain H4 | 20 | classical | Secreted |
| 9,33 | 25 | P05231 | IL6 | Interleukin-6 | 6 | classical | Secreted |
| 9,4 | 15,2 | P02788 | TRFL | Lactotransferrin | 8 | classical | Secreted |
| 12,27 | 23,0 | P02750 | A2GL | Leucine-rich alpha-2-glycoprotein | 9 | classical | Secreted |
| 5,95 | 10,8 | P18428 | LBP | Lipopolysaccharide-binding protein precursor | 4 | classical | Secreted |
| 8,36 | 48,6 | P61626 | LYSC | Lysozyme C | 6 | classical | Secreted |
| 2,06 | 4,7 | P04156 | PRIO | Major prion protein precursor | 1 | classical | Secreted |
| 2 | 10,7 | P08493 | MGP | Matrix Gla-protein precursor | 1 | classical | Secreted |
| 7,55 | 24,5 | P55058 | PLTP | Phospholipid transfer protein | 5 | classical | Secreted |
| 9,77 | 54,7 | P02753 | RETBP | Plasma retinol-binding protein precursor | 9 | classical | Secreted |
| 2,27 | 5,0 | P05121 | PAI1 | Plasminogen activator inhibitor 1 | 1 | classical | Secreted |
| 2,41 | 4,3 | P27918 | PROP | Properdin | 1 | classical | Secreted |
| 8,43 | 50,6 | P07998 | RNAS1 | Ribonuclease pancreatic | 8 | classical | Secreted |
| 2 | 5,8 | P49908 | SEPP1 | Selenoprotein P | 1 | classical | Secreted |
| 5,79 | 26,9 | P35542 | SAA4 | Serum amyloid A-4 protein | 3 | classical | Secreted |
| 4,51 | 14,4 | P02743 | SAMP | Serum amyloid P-component | 4 | classical | Secreted |
| 2,81 | 7,2 | Q7Z7G0 | TARSH | Target of Nesh-SH3 | 6 | classical | Secreted |
| 23,75 | 71,3 | P05452 | TETN | Tetranectin | 16 | classical | Secreted |
| 10,52 | 21,8 | Q9GZM7 | TINAL | Tubulointerstitial nephritis antigen-like | 7 | classical | Secreted |
| 41,64 | 56,1 | P02774 | VTDB | Vitamin D-binding protein | 29 | classical | Secreted |
| 22,77 | 52,2 | P25311 | ZA2G | Zinc-alpha-2-glycoprotein | 16 | classical | Secreted |
| 2,1 | 4,8 | Q15181 | IPYR | Inorganic pyrophosphatase | 1 | classical | Secreted |
| **OTHERS** |  |  |  |  |  |  |  |
| 5,12 | 10,5 | Q04446 | GLGB | 1,4-alpha-glucan branching enzyme | 5 | -- |  |
| 18,03 | 48,0 | Q04917 | 1433F | 14-3-3 protein eta | 12 | -- |  |
| 4 | 42,1 | P05386 | RLA1 | 60S acidic ribosomal protein P1 | 2 | -- |  |
| 5,4 | 40,9 | P05387 | RLA2 | 60S acidic ribosomal protein P2 | 3 | -- |  |
| 4,33 | 11,0 | P11766 | ADHX | Alcohol dehydrogenase class 3 chi chain | 2 | -- |  |
| 3,03 | 4,3 | O95817 | BAG3 | BAG family molecular chaperone regulator 3 | 2 | -- |  |
| 3,64 | 4,4 | P20810 | ICAL | Calpastatin | 2 | -- |  |
| 30,37 | 60,1 | P21291 | CSRP1 | Cysteine and glycine-rich protein 1 | 24 | -- | nucleus |
| 5,6 | 75,3 | P50238 | CRIP1 | Cysteine-rich protein 1 | 6 | -- |  |
| 3,04 | 18,4 | Q9BUP0 | EFHD1 | EF-hand domain-containing protein 1 | 4 | -- |  |
| 16,11 | 46,9 | P02792 | FRIL | Ferritin light chain | 14 | -- |  |
| 35,5 | 51,1 | P04075 | ALDOA | Fructose-bisphosphate aldolase A | 24 | -- |  |
| 3,01 | 30,4 | Q04760 | LGUL | Lactoylglutathione lyase | 3 | -- |  |
| 77,24 | 68,5 | P02545 | LMNA | Lamin-A/C | 50 | -- | nucleus |
| 2,02 | 6,6 | P48163 | MAOX | NADP-dependent malic enzyme | 2 | -- |  |
| 128,76 | 39,5 | Q09666 | AHNK | Neuroblast differentiation-associated protein | 96 | -- | nucleus |
| 16,42 | 58,5 | P30041 | PRDX6 | Peroxiredoxin-6 | 11 | -- |  |
| 29,83 | 53,6 | Q15124 | PGM5 | Phosphoglucomutase-like protein 5 | 20 | -- |  |
| 4,97 | 45,4 | P60903 | S10AA | Protein S100-A10 | 3 | -- | nucleus |
| 2,54 | 12,9 | P05109 | S10A8 | Protein S100-A8 | 2 | -- |  |
| 13,77 | 59,6 | P06702 | S10A9 | Protein S100-A9 | 10 | -- | nucleus |
| 6,66 | 27,9 | P37837 | TALDO | Transaldolase | 5 | -- |  |
| 2,29 | 8,9 | P12955 | PEPD | Xaa-Pro dipeptidase | 2 | -- |  |
| 2,4 | 3,5 | P41250 | SYG | Glycyl-tRNA synthetase | 1 | classical |  |
| 4,56 | 33,0 | O60888 | CUTA | Protein CutA | 3 | classical |  |
| 14,15 | 30,0 | P48745 | NOV | Protein NOV homolog precursor | 7 | classical |  |
| 2,06 | 5,2 | Q92765 | SFRP3 | Secreted frizzled-related protein 3 precursor | 1 | classical |  |
| 7,33 | 15,0 | Q8NBS9 | TXND5 | Thioredoxin domain-containing protein 5 precursor | 5 | classical |  |
| 2,01 | 9,1 | P30050 | RL12 | 60S ribosomal protein L12 | 1 | non-classical |  |
| 5,51 | 59,8 | P07108 | ACBP | Acyl-CoA-binding protein | 4 | non-classical |  |
| 4,37 | 14,6 | P04632 | CPNS1 | Calpain small subunit 1 | 3 | non-classical |  |
| 2 | 1,7 | O76074 | PDE5A | cGMP-specific 3',5'-cyclic phosphodiesterase | 1 | non-classical |  |
| 14,7 | 48,7 | Q16527 | CSRP2 | Cysteine and glycine-rich protein 2 | 8 | non-classical | nucleus |
| 12,25 | 56,7 | P52943 | CRIP2 | Cysteine-rich protein 2 | 9 | non-classical |  |
| 3,37 | 26,7 | P24534 | EF1B | Elongation factor 1-beta | 3 | non-classical |  |
| 8,87 | 24,9 | P29692 | EF1D | Elongation factor 1-delta | 6 | non-classical |  |
| 3,71 | 13,3 | P60842 | IF4A1 | Eukaryotic initiation factor 4A-I | 3 | non-classical |  |
| 3,49 | 15,1 | P09467 | F16P1 | Fructose-1,6-bisphosphatase 1 | 3 | non-classical |  |
| 14,93 | 64,8 | P09211 | GSTP1 | Glutathione S-transferase P | 13 | non-classical |  |
| 5,06 | 18,7 | P06748 | NPM | Nucleophosmin | 3 | non-classical | nucleus |
| 2 | 10,8 | P20962 | PTMS | Parathymosin | 1 | non-classical | nucleus |
| 11,52 | 77,1 | P31949 | S10AB | Protein S100-A11 | 11 | non-classical |  |
| 2,34 | 17,6 | P22061 | PIMT | L-isoaspartate(D-aspartate) O-methyltransferase | 2 | non-classical |  |
| 16,36 | 53,4 | Q15404 | RSU1 | Ras suppressor protein 1 | 12 | non-classical |  |
| 17,09 | 26,8 | P19971 | TYPH | Thymidine phosphorylase precursor | 9 | non-classical |  |
| 78,13 | 96,5 | Q01995 | TAGL | Transgelin | 93 | non-classical |  |
| 4,63 | 36,1 | O75347 | TBCA | Tubulin-specific chaperone A | 3 | non-classical |  |
| 2 | 14,6 | O43399 | TPD54 | Tumor protein D54 | 1 | non-classical |  |
| 2,56 | 37,7 | P68036 | UB2L3 | Ubiquitin-conjugating enzyme E2 L3 | 2 | non-classical |  |
| 2,02 | 8,7 | P30085 | KCY | UMP-CMP kinase | 1 | non-classical |  |
| 7,06 | 57,1 | P10599 | THIO | Thioredoxin | 4 | non-classical |  |
