# Supplementary material for: Secreted proteins from carotid endarterectomy: an untargeted approach to disclose molecular clues of plaque progression
Source: J Transl Med. 2013 Oct 16;11:260. doi: 10.1186/1479-5876-11-260 (PMC3853772; doi:10.1186/1479-5876-11-260)
Supplement: Additional file 2: Table S5 — Extra cellular identified proteins using LC- MS/MS approach. [file 1479-5876-11-260-S2.docx]

| **Paragon score** | **%Cov(95)** | **Accession** | **Entry Name** | **Name** | **Peptides(95%)** | **SecretomeP** |
| --- | --- | --- | --- | --- | --- | --- |
| **ECM ASSOCIATED** |  |  |  |  |  |  |
| 3,02 | 4,77 | P55290 | CAD13 | Cadherin-13 precursor | 2 | classical |
| 2,86 | 7,56 | Q9NQ79 | CRAC1 | Cartilage acidic protein 1 precursor | 2 | classical |
| 8,34 | 17,17 | P49747 | COMP | Cartilage oligomeric matrix protein precursor | 8 | classical |
| 45,12 | 62,88 | Q12805 | FBLN3 | EGF-containing fibulin-like extracellular matrix protein 1 precursor | 34 | classical |
| 5,53 | 1,81 | P35555 | FBN1 | Fibrillin-1 precursor | 4 | classical |
| 31,89 | 88,15 | P09382 | LEG1 | Galectin-1 | 37 | classical |
| 14,59 | 23,76 | Q08380 | LG3BP | Galectin-3-binding protein precursor | 13 | classical |
| 2,04 | 3,39 | P10915 | HPLN1 | Hyaluronan and proteoglycan link protein 1 precursor | 1 | classical |
| 4,77 | 1,62 | O15230 | LAMA5 | Laminin subunit alpha-5 precursor | 3 | classical |
| 2,01 | 1,12 | P07942 | LAMB1 | Laminin subunit beta-1 precursor | 1 | classical |
| 12,35 | 5,59 | P11047 | LAMC1 | Laminin subunit gamma-1 precursor | 7 | classical |
| 59,19 | 28,56 | Q14767 | LTBP2 | Latent-transforming growth factor beta-binding protein 2 | 42 | classical |
| 22,16 | 13,49 | P22064 | LTB1S | Latent-transforming growth factor beta-binding protein | 15 | classical |
| 10,58 | 25,52 | Q96PD5 | PGRP2 | N-acetylmuramoyl-L-alanine amidase precursor | 9 | classical |
| 0 | 26,60 | P59665 | DEF1 | Neutrophil defensin 1 precursor | 5 | classical |
| 6 | 26,60 | P59666 | DEF3 | Neutrophil defensin 3 precursor | 5 | classical |
| 2,03 | 13,33 | Q96DR8 | MUCL | Small breast epithelial mucin precursor | 1 | classical |
| 4,87 | 15,51 | P09486 | SPRC | SPARC precursor | 3 | classical |
| 2,98 | 7,38 | Q14515 | SPRL1 | SPARC-like protein 1 precursor | 3 | classical |
| 42,62 | 17,76 | P24821 | TENA | Tenascin precursor | 27 | classical |
| 8,49 | 4,64 | P22105 | TENX | Tenascin-X precursor | 8 | classical |
| 19,69 | 34,11 | Q15582 | BGH3 | Transforming growth factor-beta-induced protein ig-h3 | 16 | classical |
| 106 | 80,15 | P02647 | APOA1 | Apolipoprotein A-I precursor | 100 | classical |
| 88,05 | 14,66 | P04114 | APOB | Apolipoprotein B-100 precursor | 57 | classical |
| 54,76 | 66,92 | P06727 | APOA4 | Apolipoprotein A-IV precursor | 31 | classical |
| 47,49 | 68,99 | P02749 | APOH | Beta-2-glycoprotein 1 precursor | 35 | classical |
| 44,8 | 77,29 | P02649 | APOE | Apolipoprotein E precursor | 33 | classical |
| 41,45 | 43,43 | P10909 | CLUS | Clusterin | 30 | classical |
| 38,84 | 77,00 | P02652 | APOA2 | Apolipoprotein A-II precursor | 45 | classical |
| 13,14 | 37,04 | P05090 | APOD | Apolipoprotein D precursor | 11 | classical |
| 11,03 | 56,57 | P02656 | APOC3 | Apolipoprotein C-III precursor | 7 | classical |
| 6,28 | 35,97 | P08519 | APOA | Apolipoprotein(a) precursor (Apo(a)) (Lp(a)) | 8 | classical |
| 4,6 | 18,62 | O95445 | APOM | Apolipoprotein M | 4 | classical |
| 3,01 | 38,55 | P02654 | APOC1 | Apolipoprotein C-I precursor | 2 | classical |
| 2,67 | 7,54 | O14791 | APOL1 | Apolipoprotein-L1 precursor | 2 | classical |
| 2 | 23,76 | P02655 | APOC2 | Apolipoprotein C-II | 1 | classical |
| 192,07 | 67,53 | P01024 | CO3 | Complement C3 | 128 | classical |
| 103,31 | 46,62 | P0C0L5 | CO4B | Complement C4-B | 58 | classical |
| 11,15 | 6,74 | P01031 | CO5 | Complement C5 | 9 | classical |
| 19,32 | 23,02 | P13671 | CO6 | Complement component C6 | 13 | classical |
| 20,39 | 30,48 | P07357 | CO8A | Complement component C8 alpha chain | 18 | classical |
| 14,7 | 27,41 | P07358 | CO8B | Complement component C8 beta chain | 8 | classical |
| 8,26 | 44,06 | P07360 | CO8G | Complement component C8 gamma chain | 6 | classical |
| 29,77 | 38,82 | P02748 | CO9 | Complement component C9 | 16 | classical |
| 53,41 | 41,62 | P00751 | CFAB | Complement factor B | 36 | classical |
| 7,94 | 26,09 | P00746 | CFAD | Complement factor D | 6 | classical |
| 119,61 | 65,48 | P08603 | CFAH | Complement factor H | 88 | classical |
| 8,77 | 56,36 | Q03591 | FHR1 | Complement factor H-related protein 1 | 15 | classical |
| 3,21 | 8,18 | Q02985 | FHR3 | Complement factor H-related protein 3 | 5 | classical |
| 13,31 | 17,15 | P05156 | CFAI | Complement factor I | 10 | classical |
| 122,82 | 54,62 | P02671 | FIBA | Fibrinogen alpha chain | 134 | classical |
| 147,54 | 80,45 | P02675 | FIBB | Fibrinogen beta chain | 193 | classical |
| 73,61 | 69,98 | P02679 | FIBG | Fibrinogen gamma chain | 84 | classical |
| 32,03 | 34,01 | P01042 | KNG1 | Kininogen-1 | 21 | classical |
| 6,98 | 16,54 | P26022 | PTX3 | Pentraxin-related protein PTX3 | 4 | classical |
| 2,01 | 7,39 | P05154 | IPSP | Plasma serine protease inhibitor | 1 | classical |
| 51,71 | 40,86 | P00747 | PLMN | Plasminogen | 33 | classical |
| 3,13 | 7,71 | P35237 | SPB6 | Serpin B6 | 2 | -- |
| 2,02 | 2,78 | P78324 | SHPS1 | Tyrosine-protein phosphatase non-receptor type substrate 1 | 1 | classical |
| 4,52 | 5,92 | P07225 | PROS | Vitamin K-dependent protein S precursor | 4 | classical |
| 4,47 | 2,52 | P04275 | VWF | Von Willebrand factor | 5 | classical |
| **ECM PROTEINS** |  |  |  |  |  |  |
| 16,85 | 40,53 | P27797 | CALR | Calreticulin precursor | 11 | classical |
| 5,92 | 4,72 | P16070 | CD44 | CD44 antigen | 4 | classical |
| 20,46 | 64,84 | O43866 | CD5L | CD5 antigen-like precursor | 14 | classical |
| 10,86 | 14,00 | P02452 | CO1A1 | Collagen alpha-1(I) chain precursor | 16 | classical |
| 18,54 | 18,21 | P02461 | CO3A1 | Collagen alpha-1(III) chain precursor | 23 | classical |
| 2,01 | 1,75 | P12109 | CO6A1 | Collagen alpha-1(VI) chain precursor | 1 | classical |
| 32,45 | 18,88 | Q05707 | COEA1 | Collagen alpha-1(XIV) chain precursor | 26 | classical |
| 13,35 | 10,52 | P39059 | COFA1 | Collagen alpha-1(XV) chain precursor | 9 | classical |
| 26,38 | 19,39 | P39060 | COIA1 | Collagen alpha-1(XVIII) chain precursor | 22 | classical |
| 8,58 | 14,42 | P08123 | CO1A2 | Collagen alpha-2(I) chain precursor | 12 | classical |
| 2,06 | 1,96 | P12110 | CO6A2 | Collagen alpha-2(VI) chain precursor | 1 | classical |
| 22,93 | 8,03 | P12111 | CO6A3 | Collagen alpha-3(VI) chain precursor | 15 | classical |
| 15,07 | 11,42 | Q9Y6C2 | EMIL1 | Elastin microfibril interface-located protein 1 | 9 | classical |
| 2,26 | 1,04 | Q9BXX0 | EMIL2 | Elastin microfibril interface-located protein 2 | 1 | classical |
| 269,58 | 74,64 | P02751 | FINC | Fibronectin precursor | 258 | classical |
| 28,49 | 34,00 | P23142 | FBLN1 | Fibulin-1 precursor | 21 | classical |
| 10,85 | 8,11 | P98095 | FBLN2 | Fibulin-2 precursor | 6 | classical |
| 3,46 | 5,13 | Q9UBX5 | FBLN5 | Fibulin-5 precursor | 2 | classical |
| 3,82 | 7,62 | P18065 | IBP2 | Insulin-like growth factor-binding protein 2 precursor | 2 | classical |
| 2,38 | 6,25 | P24592 | IBP6 | Insulin-like growth factor-binding protein 6 precursor | 1 | classical |
| 19,83 | 48,09 | P10451 | OSTP | Osteopontin | 18 | classical |
| 62,95 | 50,60 | Q15063 | POSTN | Periostin precursor | 37 | classical |
| 9,75 | 23,16 | Q15113 | PCOC1 | Procollagen C-endopeptidase enhancer 1 | 6 | classical |
| 22,18 | 20,60 | P07996 | TSP1 | Thrombospondin-1 precursor | 19 | classical |
| 46,13 | 25,34 | P35442 | TSP2 | Thrombospondin-2 precursor | 26 | classical |
| 24 | 38,08 | P04004 | VTNC | Vitronectin precursor | 19 | classical |
| 8,62 | 12,12 | P08253 | MMP2 | 72 kDa type IV collagenase | 5 | classical |
| 6,5 | 11,88 | P14780 | MMP9 | Matrix metalloproteinase-9 | 6 | classical |
| 10,72 | 53,14 | P01033 | TIMP1 | Metalloproteinase inhibitor 1 | 8 | classical |
| 204,1 | 47,57 | P98160 | PGBM | Basement membrane-specific heparan sulfate proteoglycan core | 137 | non-classical |
| 57,81 | 11,07 | P13611 | CSPG2 | Versican core protein | 52 | classical |
| 39,7 | 12,22 | P16112 | PGCA | Aggrecan core protein | 27 | classical |
| 36,36 | 44,97 | P51884 | LUM | Lumican | 31 | classical |
| 30,24 | 50,54 | P21810 | PGS1 | Biglycan | 25 | classical |
| 27,56 | 46,64 | P20774 | MIME | Mimecan | 17 | classical |
| 13,03 | 4,82 | Q6UVK1 | CSPG4 | Chondroitin sulfate proteoglycan 4 | 8 | classical |
| 2,46 | 6,68 | P07585 | PGS2 | Decorin | 2 | classical |
| 2,16 | 6,27 | P35052 | GPC1 | Glypican-1 | 2 | classical |
| 2 | 4,79 | O43556 | SGCE | Epsilon-sarcoglycan | 1 | classical |
| 71,64 | 73,44 | P01009 | A1AT | Alpha-1-antitrypsin | 69 | classical |
| 25,53 | 39,01 | P01008 | ANT3 | Antithrombin-III | 22 | classical |
| 2,13 | 2,61 | P36222 | CH3L1 | Chitinase-3-like protein 1 | 1 | classical |
| 10,82 | 41,57 | P55083 | MFAP4 | Microfibril-associated glycoprotein 4 | 9 | classical |
| 5,93 | 12,30 | P51888 | PRELP | Prolargin | 5 | classical |
| 31,68 | 42,60 | P00734 | THRB | Prothrombin | 23 | classical |
